# Deletion of miR-150 Prevents Spontaneous T Cell Proliferation and the Development of Colitis

**DOI:** 10.1016/j.gastha.2023.01.021

**Published:** 2023-02-04

**Authors:** Sayaka Ishihara, Masashi Sato, Haruka Miyazaki, Haruka Saito, Tsuyoshi Sato, Noriyuki Fujikado, Satoshi Sawai, Ai Kotani, Koko Katagiri

**Affiliations:** 1 Department of Biosciences, School of Science, Kitasato University, Sagamihara, Kanagawa, Japan; 2 Department of Innovative Medical Science, School of Medicine, Tokai University, Isehara, Kanagawa, Japan; 3 Department of Immunology, School of Medicine, Kitasato University, Sagamihara, Kanagawa, Japan; 4 Discovery Immunology, Ferring Research Institute, Ferring Pharmaceuticals, San Diego, California; 5 Department of Basic Science, Graduate School of Arts and Sciences, University of Tokyo, Tokyo, Japan; 6 Division of Hematological Malignancy, Institute of Medical Sciences, Tokai University, Isehara, Kanagawa, Japan

**Keywords:** Mir-150, CD4^+^ T Cells, Colitis, Rap1

## Abstract

**Background and Aims:**

To examine the roles of microRNAs in the development of colitis, we conducted the RNA-sequencing studies using RNA derived from normal and colitogenic CD4+ T cells. Colitogenic CD4^+^ T cells demonstrated the increased expression of miR-150. We focused on the involvement of miR-150 in the colitis.

**Methods:**

We crossed miR-150 knockout mice and T-cell–specific Rap1KO mice, which is colitis model mice and spontaneously develop the colitis with tubular adenomas in microbiota-dependent manner.

**Results:**

MiR-150 silencing completely inhibited the expansion of pathogenic Th17 cells and the development of colitis.

**Conclusion:**

MiR-150 is a potential therapeutic target of inflammatory bowel diseases.

## Introduction

MicroRNAs (miRNAs) are a class of short (20–25 nucleotides) single-stranded molecules that play critical roles in the gene regulatory system. Single-stranded RNA molecules bind to an RNA-silencing complex to form a miRNA/RISC complex that can interact with target mRNA and degrade mRNA.^1^ One miRNA can regulate several targets and have multiple biological functions, such as cell proliferation and differentiation.^2^

Research has shown that miRNAs regulate immune responses.^3^, ^4^, ^5^, ^6^ Among miRNAs, miR-150 is noted for its selective expression in immature, resting B cells and T cells. It controls B cell development by targeting transcription factor c-myb.^7^ Knocking out miR-150 in mice has been shown to enhance T-cell–dependent antibody responses and increase steady-state immunoglobulins.^7^ Thus, previous articles suggest that miR-150 is inversely associated with the immunologic functions of B and T cells. On the other hand, miR-150 is believed to downregulate the suppressor of cytokine signaling (SOCS)1 and silencing miR-150 is reported to inhibit immune-mediated diseases such as experimental autoimmune encephalomyelitis and fibrosis.^8^, ^9^, ^10^ However, the role of miR-150 in autoimmune diseases remains to be completely elucidated.

Inflammatory bowel disease (IBD) is an autoimmune disease in which T cells infiltrate into large intestine lamina propria (LILP), leading to intestinal inflammation. IBD is thought to be initially mediated by pathogenic Th17 cells against microbiota.^11^, ^12^, ^13^ Previous articles reported that lymphopenia in mesenteric lymph nodes (mLN) led to the generation of colitogenic Th17 cells under the defects in Treg cells in a microbiota-dependent manner.^14^, ^15^, ^16^

Ras-related protein1 (Rap1) is a small GTPase and regulates integrin-mediated lymphocyte adhesion and migration.^17^^,^^18^ We previously reported that the homing of Rap1-deficient T cells into peripheral lymph nodes (LNs) in mice was less than one-tenth of that in wild-type (WT) T cells because of impaired arrest on high endothelial cells.^19^ Hence, T-cell–specific Rap1-knockout (Rap1KO) mice show homeostatic proliferation after lymphopenia and develop spontaneous colitis with tumors.^19^ In addition, our recent article demonstrates that pathogenic Rap1-deficient Th17 cells cause the colitis in a microbiota-dependent manner.^20^ In this study, we comprehensively examined the expression of miRNAs in pathogenic CD4^+^ cells using Rap1KO mice and found that the expression of miR-150 was increased in Rap1-deficient colitogenic T cells. We show for the first time that miR-150 deletion completely prevents the expansion of pathogenic Th17 cells and the development of spontaneous colitis using Rap1KO mice.

## Methods

### Mice and cells

All animal experiments were conducted in accordance with the Regulations for the Care and Use of Laboratory Animals at Kitasato University and the protocols used in the present study were ethically approved by the Institutional Animal Care and Use Committee at Kitasato University. *Rap*1a^f/f^ mice containing floxed exons 2–3 of *Rap1a* and *Rap1b*^f/f^ mice containing floxed exon 1 of *Rap1b* on a C57BL/6J background were maintained under specific pathogen-free conditions. These mice were crossed with CD4-Cre mice, yielding mice with T-cell–specific *Rap1a/b* deletion.^19^ miR150 knockout mice (miR-150KO) were purchased from Jackson laboratories^7^ and crossed with T-cell–specific *Rap1a/b* mice. Mice, both males and females, were used for the experiments at 6–12 weeks of ages.

CD4^+^ cells were purified from the spleen of these mice using the Naïve CD4^+^ T Cell Isolation Kit (Milteny Biotec) or sorting using MoFlo XDP (Beckman Coulter).

### Antibodies and reagents

Purified anti-CD3; purified anti-CD28; fluorescein isothiocyanate (FITC)-conjugated anti-CD3, -CD19, -B220, -F4/80, -CD64, -NK1.1, -CD62L, -Foxp3, -IFNγ; phycoerythrin (PE)- conjugated anti-CD8, -CD11c, -CD44, -Foxp3, -RORγt; phycoerythrin -cyanin 7 (PE-Cy7)-conjugated anti-CD3, -CD11b, -CD44, -IL-17A; allophycocyanin-conjugated anti-CD4, -CD44, -CD62L, -MHCⅡ, -Foxp3; Brilliant Violet 421 (BV421)-conjugated anti-CD4, -CD80 ; and Brilliant Violet 711 (BV711)-conjugated anti-CD3, -CD103 (Biolegend or e-bioscience or BD), anti-Rap1 (BD bioscience), anti-ΒΑCΗ2 (Abcam), anti-β-actin (Sigma), peroxidase-conjugated goat anti-mouse IgG, peroxidase-conjugated goat anti-rabbit IgG (Cell Signaling) were used for flow cytometry and immunoblotting.

### RNA extraction, RNA sequencing, and analysis for miRNAs

The expression profile of miRNAs was evaluated by next-generation sequencing. Total RNA was extracted from WT and Rap1-deficeint T cells stimulated with anti-CD3/28 using Trizol. Total RNA, containing the small RNA fraction, was reverse transcribed into a cDNA library using the TruSeq Small RNA Sample Prep Kit (Illumina). Analysis of changes in miRNA expression was determined with the R/Bioconductor package using RPM normalization. Statistical significance was calculated using a Student’s *t*-test to compare WT and Rap1-deficient T cells.

### Quantitative real-time RT-PCR

Total RNA was extracted from T and pro-B cell line, BAF cells with TRIzol reagent, and total RNA was purified as per the manufacturer's protocol (Invitrogen). The RNA was transcribed into cDNA with Takara PrimeScript reverse transcriptase as per the manufacturer's protocol (Takara). The expression of mature and primary miR-150-5p was measured using the Taqman MicroRNA Assays and Taqman Pri-miRNA assays (Applied Biosystems) were used for and normalized by expression of U6 or GAPH, which served as endogenous control.

### Flow cytometry and cell sorting

Immunofluorescence flow cytometry was performed as described previously.^20^ For mAb staining, the cells were washed with staining buffer (1% FBS in HBSS), resuspended in 50 μL of the same buffer, preincubated with purified anti-mouse CD16/32 (Biolegend) for 10 minutes, and incubated for 30 minutes at 4 °C with each fluorescence-conjugated mAb or isotype control matched with primary antibody. Zombie NIR dye (Biolegend) was used to assess live or dead status of cells. The samples were measured using a Gallios flow cytometry or CytoFLEX (Beckman Coulter).

### Immunoblot analysis

T cells were stimulated with immobilized anti-CD3 and anti-CD28 for the indicated times, then lysed in buffer (1% Nonidet P-40, 150 mM NaCl, 25 mM Tris-HCl [pH 7.4], 10% glycerol, 2 mM MgCl_2_, 1 mM phenylmethylsulfonylfluoride, 1 mM leupeptin, and 0.1 mM aprotinin). Cell lysates were subjected to immunoblotting.

### Histological examination

LILP sections from WT, miR-150KO, Rap1KO, and DKO mice were fixed in 10% buffered formalin and embedded in paraffin. Paraffin-embedded LILP sections were cut (6 μm), stained with hematoxylin and eosin (H&E) and examined using an Olympus IX51 light microscope equipped with CCD camera. Histological grades were assigned in a blinded manner as previously described.^19^

### Immunohistochemistry

For antigen retrieval, the slides generated from paraffin-embedded LILP sections from WT, Rap1KO, and DKO mice were deparaffinized and rehydrated to distilled water. The slides were preheated the Na-Citrate buffer (10 mM, pH 6.5) in the microwave and cooked for 10 minutes the slides in the microwave. They were blocked by endogenous peroxidase activity with 0.03% H_2_O_2_ in PBS for 30 minutes and soaked in PBS for 5 minutes, 3 times. After blocking, the sections were stained with antibodies specific for CD4 (Invitrogen) and subsequently treated with biotinylated anti-rat IgG (Vector), then developed using avidin-conjugated horseradish peroxidase with Diaminiobenzidine as substrate, using Vectastain ABC KIT (Vector). After that, the sections were counterstained with Hematoxylin.

### Induced culture of bone marrow-derived dendritic cells

Bone marrow cells were isolated and cultured in RPMI-1640 medium supplemented with 10% FBS at a density of 1 × 10^6^ cells/mL as previouly described.^20^ Subsequently, GM-CSF was added to the medium to a final concentration of 10 ng/mL. The culture medium was replaced 48 hours later to remove unattached cells and cell debris. On day 7, semi-suspended cells and loosely attached cells were collected by gently pipetting the medium against the plate. The cells were further incubated with lipopolysaccharide for 24 hours and bone marrow-derived dendritic cells were obtained. More than 90% of the cells were confirmed to be CD11c^+^ and expressed CD80.

### In vitro differentiation of CD4^+^ cells into Th17 cells

Naïve CD4^+^ cells were isolated and cultured on anti-CD3-coated and anti-CD28-coated plates with cytokines and blocking antibodies^20^; the details are as follows: 20 ng/mL rmIL-6, 2 ng/mL hTGFβ, and 1 μg/mL XMG1.2. The cells were incubated at 37 °C with 5% CO_2_ for 3–4 days before analyzing transcription factor expression.

### Intracellular cytokine staining

For the analysis of intracellular IFNγ and IL-17A, cells were stimulated for 4 hours in IMDM containing 50 ng/mL PMA, 500 ng/mL ionomycin, and 10 μg/mL brefeldin A. After surface staining, the cells were fixed, permeabilized, and intracellularly stained with the FOXP3/Transcription Factor Fixation/Permeabilization Concentrate and Diluent solution and antibody, as per the manufacturer’s protocol. Data were collected by flow cytometry.

### [^3^H]-Thymidine incorporation assay

Purified CD4^+^ cells were plated into 96-well plates in triplicates and stimulated with 0.01, 0.05, 0.5 μg/mL of anti-CD3, and 2.5 μg/mL of anti-CD28 for 24–48 hours. In total, 1 mCi [^3^H]-thymidine was added 6 hours before harvest. Labeled DNA from the cells was collected on GSC filters (Whatman) and the radioactivity was measured in a scintillation counter.

### Isolation of cells from LILP

Cells were isolated from LILP as previously reported.^20^ The intestines were cut into 1 cm pieces, placed in PBS with 30 mM EDTA and incubated at 4 °C for 15 minutes. The washed intestinal pieces were cut into 1 mm pieces and incubated for 1 hour at 37 °C in DMEM containing 1 mg/mL collagenase D and 0.2 mg/mL DNase I. The supernatants were centrifuged and resuspended in 40% Percoll (GE Healthcare). Mononuclear cells were collected from interphase of 80% and 40% Percoll after centrifugation at 2800 rpm for 15 minutes.

### Northern blot analysis

Total RNA (20 μg) was loaded and separated using SequaGel-UreaGel (national diagniostics) and transferred electrophoretically to a Nylon membrane positively charged (GE Healthcare). Membranes were UV-crosslinked. Probe (mature miR-150-5p complementary DNA) were prepared by T4 polynucleotide kinase labeling of antisense oligonucleotides with γ^32^P dATP. Hybridization was performed with UltraHyb Hybridization buffer (Ambion, Texas) at 50 °C. Blots were washed at the same temperature with 2× SSC/0.1% SDS.

### RNA-mediated interference and gene introduction via lentiviral transduction

RNA-mediated interference was used to suppress mouse Rap1a and Rap1b expression. A 19-nucleotide–specific sense RNA sequence of *Rap1a* (GAATGGCCAAGGGTTTGCA) (5′–3′) and *Rap1b* (AGACACTGATGATGTTCCA) (5′–3′) or a scrambled control RNA sequence were introduced into BAF/LFA-1 using lentivirus with a lentivirus vector with or without GFP or puromycin resistance gene (a gift from Dr Miyoshi H., RIKEN, Wako, Japan) containing the RNAi construct under control of the H1 promoter cassette, respectively. To generate expression construct of mir-150, precursor sequence of miR-150 was inserted into an *BamH1/XbaI* site of a lentivirus vector (CS-RfA-EG; a gift from H. Miyoshi, RIKEN, Wako, Japan). The production and concentration of lentivirus particles were assessed as previously described.^21^

### Statistical analysis

**Figure 1:**
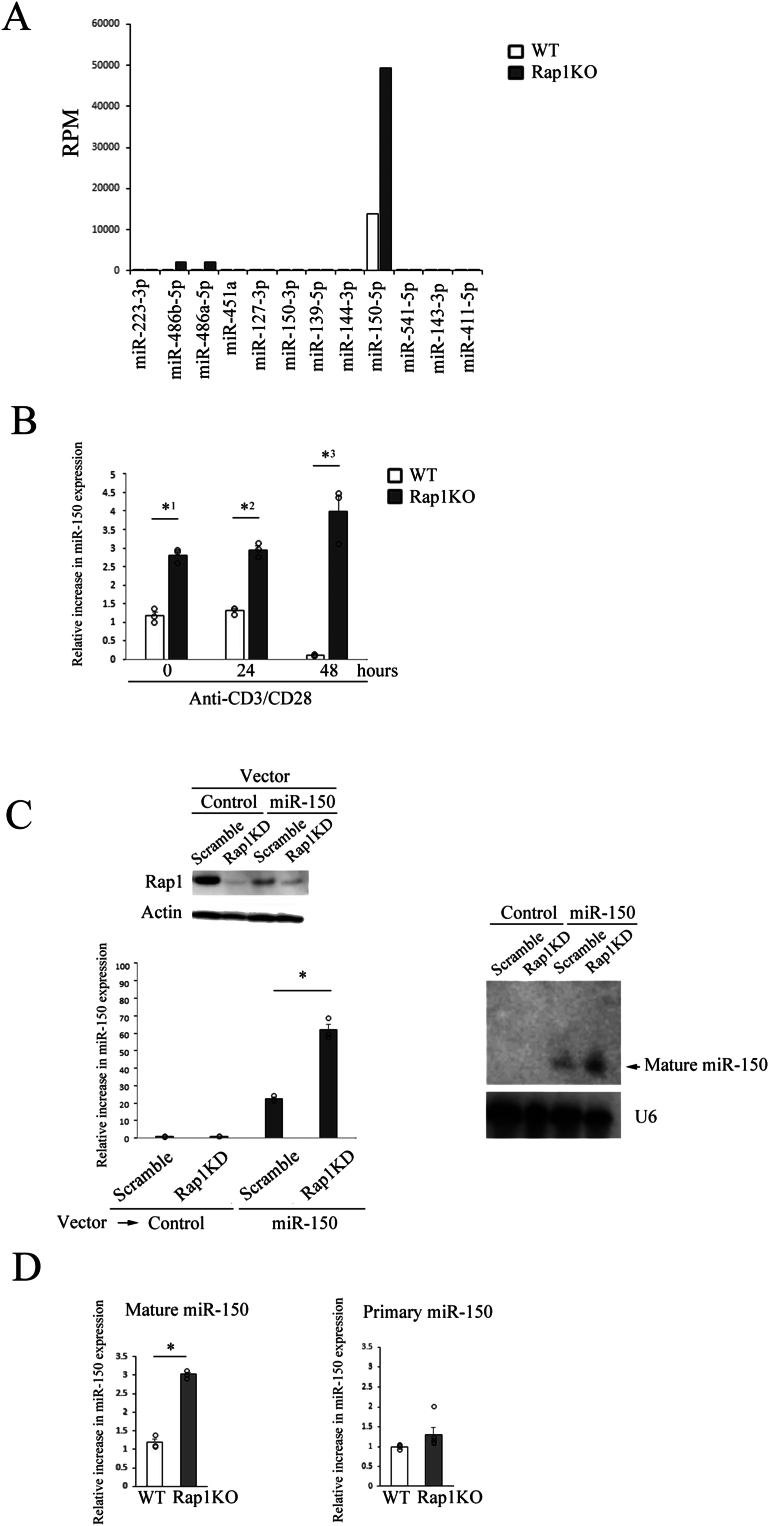
The expression of miR-150 was increased in Rap1-deficeint CD4^+^ cells. (A) Rpm (reads per megabase) of 12 miRNAs which were differentially expressed in CD4^+^ cells derived from the spleens of WT and Rap1KO mice are shown. The expression profile of miRNAs in WT and Rap1-deficeint CD4^+^ cells stimulated by the crosslinking of anti-CD3/CD28 for 2 days were detected by RNA-sequencing. (B) miR-150 expression in WT and Rap1-deficeint CD4^+^ cells at the indicated hours after the stimulation with anti-CD3/CD28 was determined in triplicate by the quantitative real-time RT-PCR (qPCR). Data represent the mean ± S.E.M. ∗^1^*P* < .001, ∗^2^*P* < .001, and ∗^3^*P* < .001 compared to WT cells. (C) (Left upper) Cell lysates from scramble and Rap1-knockdown (KD) BAF cells introduced with vector (control) and miR-150 were immunoblotted with anti-Rap1. Actin is loading control. (Left lower) miR-150 expression in scramble and Rap1-KD cells introduced with control and miR-150 expression vector was determined in triplicate by qPCR. Data represent the mean ± S.E.M. ∗*P* < .001 compared to scramble cells. (Right) Expression of mature miR-150 in RNA from scramble and Rap1-knockdown (KD) BAF cells introduced with control and miR-150 vector was determined by the northern blotting. U6 was served as loading control. (D) Mature and primary miR-150 expression in WT and Rap1-deficeint CD4^+^ cells were determined by qPCR (*n* = 3–5). Data represent the mean ± S.E.M. ∗*P* < .001 compared to WT cells.

**Figure 2:**
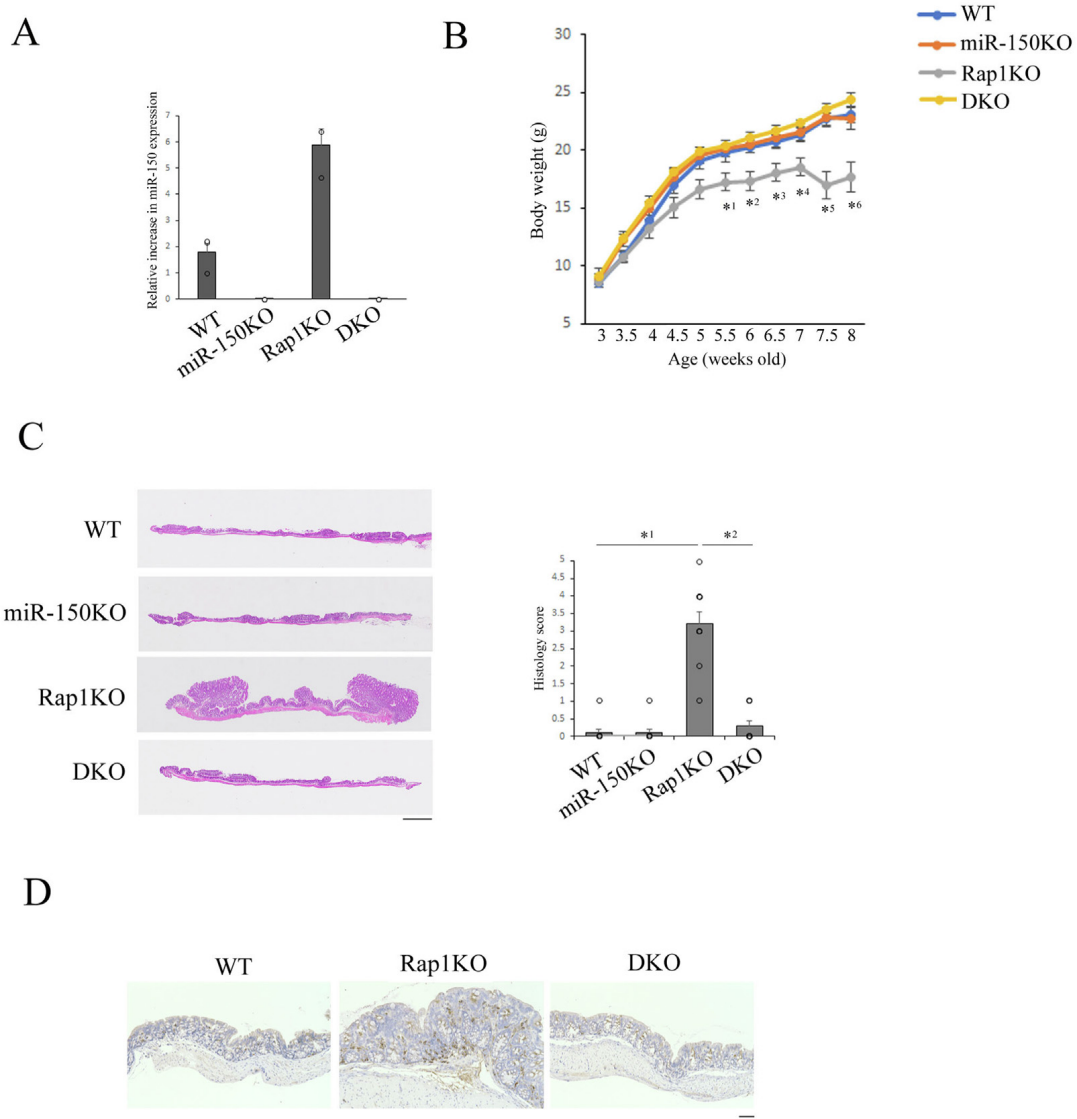
miR-150 silencing prevented the development of colitis in Rap1-deficeint mice. (A) miR-150 expression in CD4^+^ cells from WT, miR-150KO, Rap1KO and DKO mice at 8 weeks of age was determined in triplicate by qPCR. (B) The body weights of WT, miR-150KO, Rap1KO, and DKO mice (*n* = 9–11) were measured every week. Data represent the mean ± S.E.M. ∗^1^*P* < .05, ∗^2^*P* < .02, ∗^3^*P* < .03, ∗^4^*P* < .02, ∗^5^*P* < .001, and ∗^6^*P* < .002 compared with WT mice. (C) (Left) Representative histology of intestinal inflammation. Paraffin-embedded LI sections from WT, miR-150KO, Rap1KO, and DKO mice were stained with hematoxylin and eosin. Representative low (40×) magnification histological images of LI tissues are shown. Scale bars, 200 μm. (Right) Light microscopic assessment of colitis damage in WT, miR-150KO, Rap1KO, and DKO mice at 11–12 weeks of age. Data represent the mean ± S.E.M. (*n* = 10). ∗^1^*P* < .001 and ∗^2^*P* < .001 compared with Rap1KO mice. (D) Reduced CD4^+^ cells in the colon of DKO mice. LI sections from WT, Rap1KO and DKO mice at 8–9 weeks of age were stained with anti-CD4 as described in Methods. Representative high (200×) magnification immunohistochemical images of LI tissues are shown. Scale bars, 50 μm.

**Figure 3:**
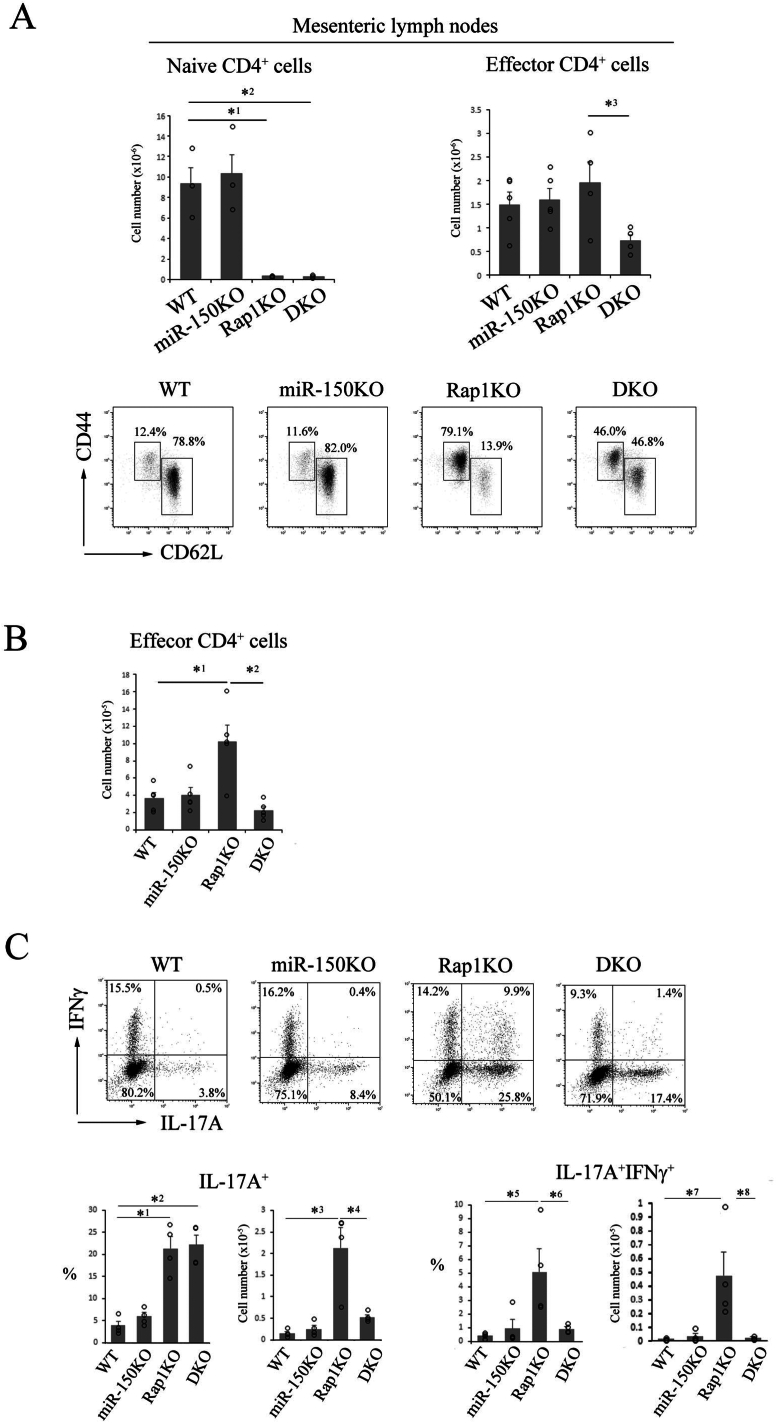
Lymphopenia in the mesenteric lymph nodes of DKO mice as same as Rap1KO mice. (A) (Upper) The numbers of naïve (left) and effector (right) CD4^+^ cells in the mesenteric lymph nodes of WT, miR-150KO, Rap1KO, and DKO mice at 8–12 weeks of age (*n* = 3–5). Data represent the mean ± S.E.M. ∗^1^*P* < .02, ∗^2^*P* < .02 compared with WT mice. ∗^3^*P* < .04 compared with Rap1KO mice. (Lower) Flow cytometry profiles of CD44 and CD62L in CD4^+^ cells from WT, miR-150, Rap1KO, and DKO mice. (B) The numbers of effector CD4^+^ cells in the LILP of WT, miR-150KO, Rap1KO and DKO mice at 8–12 weeks of age are shown. Data represent the mean ± S.E.M (*n* = 5). ∗^1^*P* < .005 compared with WT mice. ∗^2^*P* < .002 compared with Rap1KO mice. (C) (Upper) Representative IL-17A and IFNγ profiles of CD4^+^ cells from the LILP of WT, miR-150KO, Rap1KO and DKO mice at 8–12 weeks of age. CD4^+^ cells from the LILP were stimulated for 4 hours with PMA plus ionomycin. Following this, flow cytometry was performed to determine the frequency of IFNγ and IL-17A-producing cells among CD4^+^ cells. (Lower) Graphs represent the mean ± S.E.M of the ratios to CD4^+^ cells and numbers of IL-17A-expressing (left) and IFNγ + IL-17A-co-expressing (right) cells in the LILP (*n* = 4). ∗^1^*P* < .001, ∗^2^*P* < .001, and ∗^3^*P* < .001 compared with WT mice. ∗^4^*P* < .003 compared with Rap1KO mice. ∗^5^*P* < .02 and ∗^7^*P* < .02 compared with WT mice. ∗^6^*P* < .04 and ∗^8^*P* < .02 compared with Rap1KO mice.

**Figure 4:**
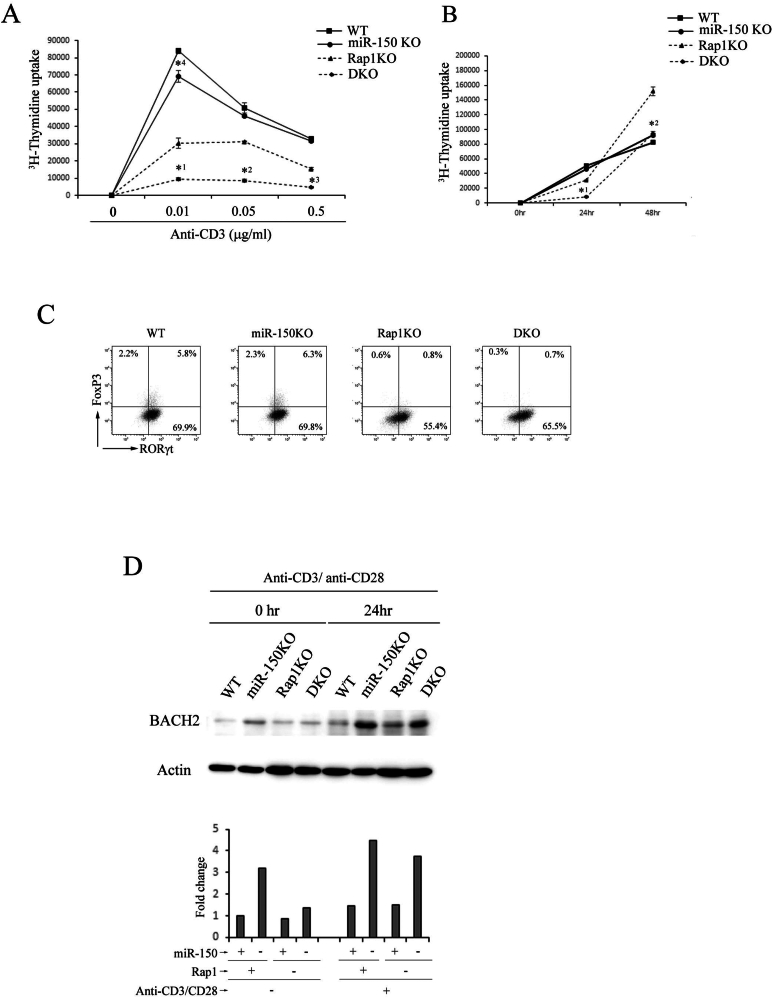
miR-150 was involved in the proliferative response of CD4^+^ cells. (A) Naïve CD4^+^ cells from WT, miR-150KO, Rap1KO, and DKO mice were stimulated with the indicated concentrations of anti-CD3 in the presence of anti-CD28 for 24 hours. [^3^H]-Thymidine uptake was measured in triplicate. Data represent the mean ± S.E.M. ∗^1^*P* < .001, ∗^2^*P* < .001, and ∗^3^*P* < .002 compared with Rap1-deficeint cells. ∗^4^*P* < .01 compared with WT cells. (B) Naïve CD4^+^ cells from WT, miR-150KO, Rap1KO, and DKO mice were stimulated with 0.5 μg/ml of anti-CD3 in the presence of anti-CD28 for 0, 24, and 48 hours. [^3^H]-Thymidine uptake was measured in triplicate. Data represent the mean ± S.E.M. ∗^1^*P* < .001, ∗^2^*P* < .001 compared with Rap1-deficeint cells. (C) Representative Foxp3 and RORγt profiles of naïve CD4^+^ cells from WT, miR-150, Rap1KO, and DKO mice; the cells were cultured for 3 days under Th17-polarizing conditions in the presence of 0.05 μg/mL of anti-CD3 and anti-CD28, subjected to flow cytometry to determine the prevalence of RORγt^+^ cells among CD4^+^ cells. (D) (Upper) Effects of miR-150 deficiency on BACH2 expression. Naïve CD4^+^ cells from WT, miR-150KO, Rap1KO, and DKO mice were stimulated with 0.5 μg/mL of anti-CD3 in the presence of anti-CD28 for 24 hours. Total lysates from the stimulated cells were immunoblotted for anti-BACH2 and actin. (Lower) Quantification of BACH2 in the experiments above, which is presented as fold change of normalized abundance of BACH2 by the total amount of actin in CD4^+^ cells before or after anti-CD3/CD28 stimulation relative to unstimulated WT CD4^+^ cells (adjusted to 1).

Statistical analysis was performed using analysis of variance followed by the Turkey-Kramer comparison test (Figures 1C, 2A–C, 3A–D, 4A and B, A2, and A3B) and two-tailed Student’s *t*-test (Figure 1B and D). *P* values less than .05 were considered significant.

## Results

### Expression of miR-150 was upregulated in Rap1-deficient CD4^+^ T cells

To investigate whether microRNAs might be involved in the development of T-cell–dependent colitis, RNA-sequencing (RNA-seq) studies were conducted using RNA derived from wild-type (WT) and Rap1-deficient CD4^+^ cells, which were stimulated by the crosslinking of anti-CD3/CD28. A list of differentially expressed miRNAs between WT and Rap1-deficient CD4^+^ cells was produced using the R/Bioconductor package (Figure 1A and Table A1). In Rap1-deficient CD4^+^ cells, the expression level of miR-150-5p was approximately 50-fold more than those of any other miRNAs in WT cells (Figure 1A). Therefore, we analyzed the expression level of miR-150 in WT and Rap1-deficient CD4^+^ cells stimulated with or without anti-CD3/CD28 by RT-PCR. As shown in Figure 1B, the expression level of miR-150 in Rap1-deficent cells was significantly higher than that of WT cells without the stimulation and it was downregulated by the stimulation with anti-CD3/CD28 in WT cells, whereas it did not decrease in Rap1-deficient cells (Figure 1B).

To confirm the effect of Rap1-deficeincy on miR-150 expression by the northern blotting, we overexpressed miR-150 in pro-B cell line, BAF cells. In the control cells, miR-150 was undetectable, whereas it was clearly detected in miR-150-expressing cells (Figure 1C). Although the overexpression of miR-150 reduced the expression of Rap1 to some extent, Rap1 knockdown upregulated the expression of mature miR-150 by northern blot and RT-PCR (Figure 1C).

We also examined the primary miR-150 expression levels in WT and Rap1-deficient CD4^+^ cells. As shown in Figure 1D, mature miR-150 expression was significantly increased in Rap1-deficient T cells compared to WT cells but there was no significant increase in the expression of primary miR-150 level by Rap1 deficiency (Figure 1D).

### Silencing miR-150 ameliorated the development of colitis

If the expression of miR-150 in CD4^+^ cells is involved in pathogenesis of colitis, it would be expected that the deletion of miR-150 could prevent the development of colitis in Rap1KO mice. To examine this possibility, we crossed miR-150 knockout mice with Rap1KO mice (double knockout mice; DKO). As shown as Figure 2A, miR-150 was not detected in the CD4^+^ cells of miR-150KO and DKO mice. Silencing miR-150 in Rap1KO mice almost completely prevented the loss of body weight (Figure 2B) and epithelial hyperplasia (Figure 2C). The increase of CD4^+^ cells in the LILP of Rap1KO mice was suppressed by miR-150 deletion (Figure 2D). These results indicate that the expression of miR-150 is necessary for the onset of colitis of Rap1KO mice.

As previously reported,^19^ Rap1 deficiency prevented the homing of naïve lymphocytes into peripheral LNs due to defective LFA-1-ICAM-1-mediated adhesion. The number of T cells in peripheral LNs of Rap1KO mice diminished to less than 10% of the level in WT mice (Figure 3A). The deletion of miR-150 did not alter the impaired homing of naïve CD4^+^ cells and DKO mice demonstrated severe peripheral CD4^+^ cell lymphopenia, as did Rap1KO mice (Figure 3A). Rap1 deficiency caused lymphopenia-induced proliferation (LIP) of naïve CD4^+^ cells, resulting in an increased proportion of effector CD4^+^ cells (Figure 3A). However, LIP was reduced in the peripheral LNs of DKO mice (Figure 3A), suggesting that miR-150 facilitates LIP.

Consistent with that, an increase in the number of effector CD4^+^ cells in the LILP of Rap1KO mice was suppressed in the LILP of DKO mice (Figure 3B). As previously reported,^22^ the numbers and ratios of IL-17^+^ and IL-17^+^ IFNγ^+^ cells were significantly increased in the LILP of Rap1KO mice (Figure 3C). On the other hand, the ratio of IL-17^+^ cells was not reduced but the number of IL-17^+^ cells was significantly reduced in the LILP of DKO mice compared to Rap1KO mice (Figure 3C). Both the ratio and the number of IL-17^+^ IFNγ^+^ cells were significantly diminished in the LILP of DKO mice compared to Rap1KO mice (Figure 3C), suggesting that the generation of IL-17^+^ IFNγ^+^ cells might be induced by severe inflammation. There was no difference in apoptosis of effector CD4^+^ cells and Th17 cells derived from LILP and mLN between WT, miR-150KO, Rap1KO, and DKO mice (Figure A1A). Our previous article demonstrated that RORγt^+^ Treg cells were reduced in the LILP of Rap1 KO mice. RORγt^+^ Treg cells were not recovered in the LILP of DKO mice (Figure A1B). These data suggest that miR-150 ablation might directly suppress the expansion of pathogenic Th17 cells.

In addition, we examined the effects of miR-150 deficiency on the production of cytokines such as IL-6 in dendritic cells (DC), which is reported to be important for LIP.^23^ However, miR-150 ablation did not affect the subsets of dendritic cells in the mLN and their CD80 expression (Figure A2A). As shown in Figure A2B, cytokine production of bone marrow–derived dendritic cells from miR-150-deficient mice was normal. These results suggest that the inhibition of LIP by miR-150 deletion is not due to the reduced production of IL-6 by dendritic cells.

### MiR-150 inhibited the proliferation of CD4^+^ cells in response to the crosslinking of anti-CD3 and anti-CD28

We examined the proliferative responses of naïve CD4+ cells derived from WT, miR-150KO, Rap1KO, and DKO mice to various concentrations (0.01–0.5 μg/mL) of anti-CD3 in the presence of anti-CD28. As previously reported, Rap1-deficient naïve CD4+ cells proliferated less than WT cells at 24 hrs after stimulation (Figure 4A). The miR-150 deficiency significantly suppressed the proliferation of Rap1-deficient cells at 24 hours after the stimulation (Figure 4A). As previously reported, Rap1-deficient T cells proliferated more than WT cells at 48 hours after stimulation with a higher dose (>0.05 mg/mL) of anti-CD3 (Figure 4B). The miR-150 deficiency significantly inhibited the proliferation of Rap1-deficeint cells at 48 hours after the stimulation (Figure 4B). On the other hand, when the cells were cultured under a Th17-polarizing condition, silencing miR-150 did not affect RORγt expression in WT and Rap1-deficient naïve CD4+ cells (Figure 4C). These data suggest that miR-150 facilitates the proliferation of Rap1-deficient naïve CD4^+^ cells but does not affect the differentiation into Th17 cells.

BTB and CNC Homology 1, Basic Leucine Zipper Transcription Factor 2 (BACH2) is critical for the regulation of T cell proliferation and maintenance of the naïve T cell state.^24^^,^^25^ A previous article reported that BACH2 was a potential target of miR-150.^26^ Therefore, we examined the effects of miR-150 deficiency on the expression of BACH2. As shown in Figures 4D and A3, BACH2 expression in miR-150-deficient CD4^+^ cells was increased in WT and Rap1-deficient cells with the stimulation with anti-CD3/anti-CD28, although there was no difference in the expression of BACH2 between WT and Rap1-deficient cells. We could not detect the expression of SOCS1 in all kinds of CD4^+^ cells at 24 hours after stimulation with anti-CD3/CD28. These results suggest that BACH2 is one of the target candidates of miR-150 involved in the prevention of LIP and colitis.

## Discussion

Our previous articles reported that Rap1 deficiency caused lymphopenia in mLN and the reduction in the differentiation of RORγt^+^ Treg, which led to the expansion of Th17 cells in the LILP and development of colitis.^19^^,^^20^ In the present study, we found that miR-150 ablation prevents the expansion of Th17 cells and development of colitis in Rap1KO mice. However, miR-150 deficiency ameliorated neither the reduced number of naïve CD4+ cells in mLNs nor defective differentiation into RORγt^+^ Treg cells in Rap1KO mice. The deletion of miR-150 suppressed the LIP and the proliferative responses of Rap1-deficient CD4^+^ cells to anti-CD3/28, suggesting that miR-150 might be directly involved in the spontaneous proliferation of colitogenic T cells.

Previous study reports that silencing miR-150 ameliorates experimental autoimmune encephalomyelitis (EAE).^8^ EAE is a model for multiple sclerosis (MS), which is an autoimmune disease and is mediated by pathogenic T cells against myelin antigens.^27^ The deletion of miR-150 suppressed EAE-mediated upregulation of CD4^+^ and CD8^+^ T cells and reduced the expression of cytokines such as IL-6 and TNF-α in the spleen and spinal cord after EAE induction, suggesting that the deletion of miR-150 may suppress the expansion of pathogenic T cells.^8^ Thus, the inhibitors of miR-150 may be useful for treating various autoimmune diseases by suppressing the expansion of pathogenic T cells.

The role of miR-150 in controlling immunity has been demonstrated in different types of immune cells such as B, Natural killer (NK), NK T (NKT), and CD8^+^ T cells.^28^, ^29^, ^30^ It is reported that miR-150 plays important roles in B cell development via targeting Myb, thereby the knockout of miR-150 promotes the expansion of B1 cells and enhanced humoral immune responses.^7^ It is also reported that miR-150 is involved in CD8^+^ cell differentiation via targeting forkhead box O1 (Foxo1) and the deletion of miR-150 accelerated the differentiation of memory CD8^+^ T cell formation.^31^ On the other hand, in this study, miR-150 is highly expressed in naïve CD4^+^ cells and decreased after the stimulation with anti-CD3/CD28 and the knockout of miR-150 inhibited the expansion of Th17 cells in LILP of Rap1-deficient mice. In addition, the proliferation of Rap1-deficient CD4^+^ cells in response to anti-CD3/CD28 was significantly reduced by the deletion of miR-150. Therefore, it is likely that miR-150 targets the suppressor of CD4^+^ cell proliferation.

Bach2 is a leucine zipper transcription factor and is reported to play important roles in T cell proliferation and differentiation and B cell development.^24^ Bach2 suppresses effector-related genes to maintain the naïve state of T cells.^25^ In inflammatory diseases, Bach2 is often downregulated in CD4^+^ T cells and negatively associated with the disease severity.^32^ A recent article reported that miR-150 promotes T cell activation in severe aplasic anemia by targeting Bach2 and miR-150 inhibitor suppressed the disease progression.^26^ In this study, we found that Bach2 expression was increased in CD4^+^ cells from miR-150KO mice with or without the stimulation by anti-CD3/CD28. Bach2 might be one of targets of miR-150, which prevents the LIP and expansion of colitogenic Th17 cells in Rap1KO mice.

It has been reported that miR-150 promotes maturation and functions of immune cells by targeting SOCS 1. In a renal ischemia model, miR-150 is reported to promote renal fibrosis by downregulating SOCS 1 in kidney resident cells.^9^ The knockout of miR-150 or administration of miR-150 inhibitors attenuates tubulointerstitial fibrosis through the SOCS1/Janus kinase (JAK) and signal transducer and activators of transcription (STAT) pathway.^10^ In this study, there was no difference in cytokine production by dendritic cells between WT and miR-150KO mice. However, miR-150 KO mice which we used in this study were systemically knocked out miR-150, so we could not exclude the effects of miR-150 ablation on nonimmune cells such as fibroblast, stromal, or epithelial cells to prevent the LIP and the expansion of Th17 cells.

The expression of miR-150 in naïve CD4^+^ cells from Rap1KO mice was significantly increased. The expression level of miR-150 was reduced in WT CD4^+^ cells after the stimulation with anti-CD3/CD28, whereas it was not reduced in Rap1-deficient CD4^+^ cells. Our previous article demonstrates that Rap1 is critical for TCR-distal signaling such as nuclear translocation of nuclear factor of activated T-cells (NFAT); thus, defective signaling might cause impaired reduction of miR-150 in Rap1-deficient cells.^20^ On the other hand, a previous article reported that Rap1 binds a sequence in the stem-loop precursor of miR-124 and restricts its transcription.^33^ In this study, Rap1 ablation did not affect the expression level of primary miR-150, suggesting that Rap1 possibly participates in the processing or degradation of miR-150.

## Conclusion

In this study, we show that an miR-150 inhibitor might be a potential therapeutic agent for IBD. However, the exact mechanism by which miR-150 prevents the colitis in Rap1KO mice is not completely elucidated. Clarifying the targets of miR-150 using DKO mice may lead to discovery of a novel therapy for IBD.

## Supplementary materials

Table A1

Figure A1

Figure A2

Figure A3
