# Supplementary material for: Deletion of miR-150 Prevents Spontaneous T Cell Proliferation and the Development of Colitis
Source: Gastro Hep Adv. 2023 Feb 4;2(4):487–96. doi: 10.1016/j.gastha.2023.01.021 (PMC11308117; doi:10.1016/j.gastha.2023.01.021)
Supplement: Table A1 [file mmc1.pdf]

Table A1 RPM, *m* .value, *p* -value in WT and Rap1KO CD4$^+$ cells.

	RPM			
	WT	KO	*m*.value	*p*.value
mmu-miR-223-3p	2.166427096	86.38302469	6.193114629	0.001266894
mmu-miR-486b-5p	294.634085	2056.270178	3.67878998	0.009830713
mmu-miR-486a-5p	296.0783698	2060.008851	3.674355923	0.009886798
mmu-miR-451a	11.91534903	86.97334149	3.74350844	0.012823216
mmu-miR-127-3p	95.6838634	3.541900785	-3.879923548	0.013644086
mmu-miR-150-3p	36.10711827	223.9268385	3.508428653	0.013858791
mmu-miR-139-5p	34.30176235	210.5463244	3.493539473	0.014239833
mmu-miR-144-3p	4.332854192	30.89324573	3.709658248	0.022318044
mmu-miR-150-5p	13935.18122	49418.3707	2.702072071	0.035227271
mmu-miR-541-5p	34.66283353	1.574178127	-3.584962501	0.03666265
mmu-miR-143-3p	3.610711827	20.66108791	3.392317423	0.040614553
mmu-miR-411-5p	33.2185488	1.770950392	-3.353636955	0.046640208
mmu-miR-10a-5p	126.0138427	375.6382554	2.451518161	0.052134106
mmu-miR-34a-3p	12.27642021	0.196772266	-5.087462841	0.053000631
mmu-miR-203-3p	101.0999311	296.9293491	2.430094074	0.0543673
mmu-miR-378d	296.8005121	842.7756145	2.381411378	0.055596045
mmu-miR-1983	135.0406223	12.59342501	-2.54689446	0.0565795
mmu-miR-181a-5p	27993.12665	77592.61726	2.346603144	0.056946389
mmu-miR-144-5p	3.610711827	17.51273166	3.153805336	0.057322975
mmu-miR-26a-5p	17150.5201	46713.53913	2.321344812	0.058944676
mmu-miR-223-5p	0.722142365	7.477346101	4.247927513	0.062939789
mmu-let-7c-5p	1430.564026	3750.085842	2.266095941	0.063835889
mmu-let-7b-3p	15.52606085	47.2253438	2.480625841	0.069310296
mmu-miR-409-3p	19.13677268	0.983861329	-3.40599236	0.072499291
mmu-miR-181c-5p	3947.59124	9656.402173	2.166269541	0.072874187
mmu-miR-298-5p	6740.837909	817.3919922	-2.168071531	0.072914825
mmu-miR-155-3p	323.5197797	37.58350277	-2.229926094	0.073740476
mmu-miR-1843a-5p	249.5001872	598.5812326	2.138262537	0.077758864
mmu-miR-365-3p	32.85747762	2.558039456	-2.807354922	0.077820351
mmu-miR-26b-3p	23.83069806	63.36066959	2.286522759	0.080555986
mmu-let-7b-5p	286.3294478	651.1194276	2.061002521	0.086091187
mmu-miR-28a-5p	402.2332975	904.1685614	2.044315629	0.087436167
mmu-miR-29a-3p	3053.578992	6794.349566	2.029590669	0.087813323
mmu-miR-7683-5p	17.33141677	0.983861329	-3.263034406	0.087990785
mmu-miR-322-5p	63.18745696	146.0050212	2.084064265	0.090800346

mmu-miR-378c	2294.968437	4989.554344	1.99619304	0.091973918
mmu-let-7g-5p	9999.866404	21538.29867	1.982679958	0.093450048
mmu-miR-155-5p	11040.47355	1523.607654	-1.981482319	0.093780989
mmu-miR-26b-5p	4135.709326	8817.365231	1.967969218	0.0954869
mmu-miR-200a-3p	13.72070494	34.43514652	2.203283598	0.105916371
mmu-miR-92b-3p	96.40600577	201.8883447	1.942119084	0.106269155
mmu-miR-300-3p	8.304637201	0.196772266	-4.523561956	0.107459647
mmu-miR-1839-5p	571.214611	1141.279142	1.8743033	0.109643639
mmu-miR-34a-5p	190.6455844	26.95780042	-1.946362036	0.112734011
mmu-miR-28a-3p	99.29457523	197.3625826	1.866818082	0.11746958
mmu-miR-215-5p	2.166427096	8.854751962	2.906890596	0.119835855
mmu-miR-3964	112.654209	215.2688588	1.809994804	0.125839883
mmu-miR-210-5p	424.2586396	66.70579811	-1.793303578	0.128090109
mmu-miR-30a-5p	613.4599393	1112.353619	1.734330844	0.1324252
mmu-miR-341-3p	7.221423653	0.196772266	-4.321928095	0.134765891
mmu-miR-381-3p	7.221423653	0.196772266	-4.321928095	0.134765891
mmu-miR-125a-5p	91.35100921	169.6176931	1.768550484	0.134970368
mmu-miR-10b-5p	5.777138922	15.545009	2.303780748	0.135573773
mmu-miR-1843b-5p	178.3691642	323.2968327	1.733749533	0.136362137
mmu-miR-6240	72.21423653	10.23215782	-1.943416472	0.136537866
mmu-miR-29a-5p	4.693925375	13.38051408	2.387023123	0.136728927
mmu-miR-212-5p	171.5088118	26.76102815	-1.804320862	0.136732952
mmu-miR-101a-3p	1717.615616	3028.915488	1.694150501	0.138765359
mmu-miR-410-3p	11.91534903	0.787089063	-3.044394119	0.141478398
mmu-miR-450a-5p	5.054996557	13.77405861	2.321928095	0.142483126
mmu-miR-296-5p	96.04493459	14.56114767	-1.84582907	0.143016428
mmu-miR-6236	6.86035247	0.196772266	-4.247927513	0.145879265
mmu-miR-322-3p	6.86035247	16.92241486	2.178337241	0.146135067
mmu-miR-30b-5p	1056.133209	1782.953501	1.631233655	0.151489651
mmu-miR-7688-5p	20.58105741	2.164494924	-2.373458396	0.153639529
mmu-miR-200b-3p	22.74748451	43.09312621	1.797507136	0.15798199
mmu-miR-181b-5p	774.858758	1273.11656	1.592115636	0.160092843
mmu-miR-540-3p	7.943566018	0.393544532	-3.459431619	0.164932483
mmu-miR-142a-5p	138376.92	222165.5299	1.558788047	0.165892397
mmu-let-7a-5p	10991.72894	17485.57708	1.545503467	0.168986565
mmu-miR-449c-5p	150.9277544	245.5717877	1.578043087	0.169367833
mmu-miR-141-3p	183.4241608	296.3390323	1.567821368	0.170326659
mmu-miR-6899-3p	0	2.558039456	3.700439718	0.172095015
mmu-miR-3473a	11.91534903	0.983861329	-2.722466024	0.172635048

mmu-miR-363-5p	81.96315846	13.77405861	-1.69726547	0.176131579
mmu-miR-378b	32.13533526	55.29300669	1.658692889	0.176970424
mmu-miR-3473b	31.77426407	4.525762114	-1.935869663	0.182599063
mmu-miR-1199-5p	0.722142365	3.935445316	3.321928095	0.190513007
mmu-miR-101a-5p	4.332854192	10.42893009	2.142957954	0.193625338
mmu-miR-132-5p	94.96172104	17.31595939	-1.579487371	0.196674957
mmu-miR-130b-5p	819.9926558	164.304842	-1.443479603	0.198157361
mmu-miR-29c-3p	84.12958556	128.098745	1.482327589	0.19913806
mmu-miR-211-5p	59.57674514	91.69587587	1.49786393	0.201119092
mmu-miR-362-3p	28.16355225	45.84793793	1.578783926	0.201151118
mmu-miR-365-2-5p	8.304637201	0.590316797	-2.938599455	0.201762957
mmu-miR-5128	36.82926063	5.903167974	-1.765534746	0.204126116
mmu-miR-192-5p	3162.622489	4522.613757	1.391791824	0.207902908
mmu-miR-194-5p	130.7077681	191.6561869	1.427932075	0.208220651
mmu-miR-5113	1.444284731	5.116078911	2.700439718	0.208469392
mmu-miR-542-3p	4.693925375	10.42893009	2.027480736	0.212774583
mmu-miR-3970	4.332854192	9.838613291	2.058893689	0.213726233
mmu-miR-339-3p	68.24245352	100.3538556	1.432111013	0.216391855
mmu-miR-3969	26.35819633	41.32217582	1.524420959	0.218432469
mmu-miR-7015-3p	6.86035247	13.38051408	1.839535328	0.222597917
mmu-miR-147-3p	18.7757015	30.30292894	1.566346823	0.222883066
mmu-miR-101c	26.71926752	41.32217582	1.504792152	0.223391866
mmu-miR-199a-3p	0.361071183	2.754811721	3.807354922	0.223722398
mmu-miR-199b-3p	0.361071183	2.754811721	3.807354922	0.223722398
mmu-miR-29b-3p	114.0984937	160.9597134	1.372176285	0.225664622
mmu-miR-15a-5p	808.7994491	1108.811718	1.330916878	0.2268041
mmu-miR-378a-3p	14957.73481	20147.11875	1.305438211	0.232707309
mmu-miR-129-5p	3.610711827	0	-3.321928095	0.233851149
mmu-miR-375-3p	0	1.967722658	3.321928095	0.233851149
mmu-miR-5120	6.138210105	0.393544532	-3.087462841	0.239259863
mmu-miR-130a-3p	23.46962687	3.73867305	-1.7744403	0.240315825
mmu-miR-212-3p	467.9482527	107.2408849	-1.249737583	0.258655068
mmu-miR-504-5p	45.8560402	9.051524227	-1.465122731	0.260202001
mmu-miR-664-3p	18.41463032	27.15457268	1.436099115	0.263030832
mmu-miR-8111	5.054996557	9.641841025	1.807354922	0.263844797
mmu-miR-1187	6.86035247	0.590316797	-2.662965013	0.264686775
mmu-miR-99b-5p	75.82494836	99.5667665	1.268748057	0.264849839
mmu-miR-6992-5p	4.332854192	0.196772266	-3.584962501	0.269673902
mmu-let-7e-5p	71.49209417	92.87650946	1.253286429	0.271412034

mmu-miR-181d-5p	413.4265041	516.5271978	1.196969824	0.272259884
mmu-miR-32-5p	117.3481344	149.3501498	1.223660167	0.273091065
mmu-miR-326-3p	28.16355225	38.5673641	1.329307625	0.275848354
mmu-miR-140-5p	71.85316535	92.0894204	1.233740099	0.278303988
mmu-miR-466b-3p	330.7412033	78.70890633	-1.195347598	0.280681111
mmu-miR-466c-3p	330.7412033	78.70890633	-1.195347598	0.280681111
mmu-miR-466p-3p	330.7412033	78.70890633	-1.195347598	0.280681111
mmu-miR-466e-3p	330.7412033	78.90567859	-1.191745362	0.281940435
mmu-miR-1957a	9.026779566	14.3643754	1.545968369	0.282411462
mmu-miR-181a-1-3p	243.7230483	299.4873886	1.173008952	0.283949285
mmu-miR-8097	1.083213548	3.541900785	2.584962501	0.284076578
mmu-miR-149-5p	43.6896131	56.67041255	1.251061764	0.285659334
mmu-miR-1839-3p	31.05212171	41.12540356	1.281094377	0.288105174
mmu-miR-434-5p	2.888569461	0	-3	0.291535277
mmu-miR-3473e	14.80391849	2.36126719	-1.772589504	0.29330651
mmu-miR-18b-3p	59.57674514	13.18374181	-1.300233024	0.293370626
mmu-miR-93-5p	7441.316003	8812.249152	1.119708889	0.29672298
mmu-miR-466a-3p	295.7172986	72.60896609	-1.150242636	0.298198721
mmu-miR-30a-3p	34.66283353	44.47053207	1.235216462	0.300081601
mmu-miR-330-3p	16.24820322	2.754811721	-1.684498174	0.301346934
mmu-miR-3968	534.0242791	133.4115962	-1.125264874	0.301703408
mmu-miR-140-3p	2779.164893	3257.564861	1.104898858	0.302756615
mmu-miR-877-3p	49.82782321	11.01924689	-1.301169535	0.303467932
mmu-miR-6395	0.722142365	2.754811721	2.807354922	0.305341368
mmu-let-7f-5p	61782.16792	71990.31408	1.096368706	0.305428567
mmu-miR-449a-3p	2.527498279	5.312851177	1.94753258	0.316734605
mmu-miR-34c-5p	30.69105053	6.493484772	-1.364996817	0.31906673
mmu-miR-151-5p	461.8100426	528.530306	1.070443041	0.320025028
mmu-miR-874-3p	13.35963376	18.29982072	1.329705445	0.322337904
mmu-miR-434-3p	6.86035247	0.787089063	-2.247927513	0.322487866
mmu-miR-26a-2-3p	9.387850749	13.57728634	1.408084739	0.325498259
mmu-miR-129-2-3p	2.527498279	0	-2.807354922	0.327219494
mmu-miR-3960	2.527498279	0	-2.807354922	0.327219494
mmu-miR-7045-3p	2.527498279	0	-2.807354922	0.327219494
mmu-let-7d-5p	2592.852163	2913.213395	1.043827823	0.327576666
mmu-miR-1306-5p	49.46675202	11.60956368	-1.215389034	0.332271137
mmu-miR-1843b-3p	43.32854192	51.55433364	1.126532406	0.333256067
mmu-miR-320-3p	260.6933939	293.1906761	1.045241588	0.333511576
mmu-miR-532-5p	801.9390967	888.4267802	1.023516595	0.337716032

mmu-miR-6537-3p	18.05355913	3.541900785	-1.473931188	0.341558305
mmu-miR-132-3p	852.1279911	230.6170955	-1.00981429	0.345800546
mmu-miR-700-3p	14.44284731	18.69336525	1.247927513	0.346921429
mmu-miR-1955-3p	2.527498279	4.919306645	1.836501268	0.350998442
mmu-miR-296-3p	165.3706017	44.07698754	-1.031848866	0.354898274
mmu-miR-142a-3p	8180.067643	8769.352798	0.976113988	0.356170506
mmu-miR-339-5p	150.5666832	164.304842	1.001728814	0.357564767
mmu-miR-1249-3p	15.16498967	19.08690978	1.207595419	0.358969901
mmu-miR-23a-3p	379.8468842	405.7444121	0.970909628	0.363557513
mmu-miR-879-5p	3.249640644	0.196772266	-3.169925001	0.364302468
mmu-miR-345-3p	111.5709954	29.71261214	-1.033058289	0.36478505
mmu-miR-350-5p	23.10855569	27.35134495	1.118941073	0.366240147
mmu-miR-378a-5p	59.93781632	66.31225358	1.02156535	0.366990201
mmu-miR-1943-5p	21.30319978	25.38362229	1.128584206	0.367228061
mmu-miR-18b-5p	149.4834696	40.53508676	-1.00698643	0.367751392
mmu-miR-23a-5p	0	1.180633595	2.584962501	0.368661849
mmu-miR-3109-3p	0	1.180633595	2.584962501	0.368661849
mmu-miR-338-3p	0	1.180633595	2.584962501	0.368661849
mmu-miR-134-5p	2.166427096	0	-2.584962501	0.368661849
mmu-miR-666-5p	2.166427096	0	-2.584962501	0.368661849
mmu-miR-7674-5p	2.166427096	0	-2.584962501	0.368661849
mmu-miR-181b-1-3p	6.138210105	8.854751962	1.404390255	0.373581978
mmu-miR-450b-5p	10.83213548	13.97083087	1.242856524	0.37395684
mmu-miR-16-2-3p	61.38210105	67.09934264	1.004236993	0.374012902
mmu-miR-3535	106.5159989	114.1279142	0.975337946	0.374586112
mmu-miR-30d-3p	336.5183422	353.7965339	0.947991161	0.374724634
mmu-miR-30e-3p	809.5215915	845.5304262	0.938543499	0.375600061
mmu-miR-25-3p	13595.41324	14142.41629	0.932665017	0.375954259
mmu-miR-7655-5p	6.86035247	0.983861329	-1.925999419	0.378605126
mmu-miR-421-5p	11.55427784	2.164494924	-1.540568381	0.38180853
mmu-miR-96-5p	10.83213548	13.77405861	1.222392421	0.382140674
mmu-miR-31-3p	5.054996557	0.590316797	-2.222392421	0.382623327
mmu-miR-222-3p	327.4915627	93.46682626	-0.933175037	0.387326016
mmu-miR-24-2-5p	188.4791573	195.7884045	0.930646719	0.387487346
mmu-miR-1843a-3p	6.138210105	8.46120743	1.338801913	0.398328608
mmu-miR-350-3p	14.44284731	17.11918713	1.121015401	0.398656944
mmu-miR-22-3p	15646.65863	15662.48204	0.877214644	0.402621516
mmu-miR-361-3p	219.8923502	222.7462049	0.894359825	0.40343477
mmu-let-7i-5p	26270.09497	26229.93981	0.87354947	0.404383198

mmu-miR-203-5p	0.361071183	1.574178127	3	0.405013114
mmu-miR-335-3p	0.361071183	1.574178127	3	0.405013114
mmu-miR-1945	2.888569461	0.196772266	-3	0.405013114
mmu-miR-505-5p	7.943566018	1.377405861	-1.652076697	0.411555755
mmu-miR-301b-3p	152.3720391	44.27375981	-0.907317997	0.412130033
mmu-miR-582-3p	4.693925375	0.590316797	-2.115477217	0.413658936
mmu-miR-1948-3p	1.083213548	2.558039456	2.115477217	0.413658936
mmu-miR-574-5p	4.332854192	6.296712506	1.415037499	0.415087273
mmu-miR-6399	10.83213548	2.164494924	-1.447458977	0.415324179
mmu-miR-28c	3.971783009	5.903167974	1.447458977	0.415324179
mmu-let-7f-2-3p	76.90816191	78.31536179	0.901915	0.416058553
mmu-miR-27a-3p	1352.933721	1330.574061	0.851714039	0.41682839
mmu-miR-466o-3p	1.805355913	0	-2.321928095	0.417059097
mmu-miR-667-3p	1.805355913	0	-2.321928095	0.417059097
mmu-miR-6992-3p	1.805355913	0	-2.321928095	0.417059097
mmu-miR-7653-3p	1.805355913	0	-2.321928095	0.417059097
mmu-miR-103-2-5p	0	0.983861329	2.321928095	0.417059097
mmu-miR-147-5p	0	0.983861329	2.321928095	0.417059097
mmu-miR-196b-5p	0	0.983861329	2.321928095	0.417059097
mmu-miR-18a-3p	290.3012309	86.77656922	-0.866416846	0.420769752
mmu-miR-361-5p	292.1065868	286.3036468	0.846807545	0.424928817
mmu-miR-652-3p	305.1051493	298.8970718	0.846098623	0.424990724
mmu-miR-219a-5p	16.97034558	18.89013752	1.030373649	0.425793906
mmu-miR-744-3p	22.02534214	23.80944416	0.9881259	0.42705011
mmu-miR-3098-3p	13.35963376	2.951583987	-1.30256277	0.428144369
mmu-miR-7650-5p	6.138210105	0.983861329	-1.765534746	0.429654779
mmu-miR-3473d	216.6427096	65.32839225	-0.853779259	0.431237846
mmu-miR-30c-2-3p	2.527498279	4.132217582	1.584962501	0.434570297
mmu-miR-30d-5p	17687.43295	16909.23112	0.81084277	0.436515522
mmu-miR-31-5p	582.7688888	179.6530787	-0.821953813	0.436969473
mmu-miR-106a-5p	331.8244169	101.7312614	-0.829900581	0.437589092
mmu-miR-674-3p	177.2859507	53.71882857	-0.846822073	0.438290452
mmu-miR-342-5p	294.2730139	281.3843401	0.811143183	0.443405244
mmu-miR-674-5p	57.77138922	16.72564259	-0.912537159	0.44412124
mmu-miR-449a-5p	335.7961999	318.5742984	0.799800364	0.448551333
mmu-miR-33-3p	16.6092744	17.90627619	0.984232684	0.448625655
mmu-miR-181c-3p	212.6709266	202.085117	0.802096642	0.45088416
mmu-miR-6997-5p	2.527498279	0.196772266	-2.807354922	0.451749967
mmu-miR-7672-5p	2.527498279	0.196772266	-2.807354922	0.451749967

mmu-miR-15a-3p	28.16355225	28.72875081	0.90442234	0.452613272
mmu-miR-664-5p	25.27498279	25.97393909	0.915111102	0.45344545
mmu-miR-6516-5p	3.249640644	4.72253438	1.415037499	0.456531955
mmu-miR-486a-3p	1.805355913	3.148356253	1.678071905	0.458346555
mmu-miR-191-5p	70776.45108	22647.89748	-0.768135911	0.45940745
mmu-miR-16-5p	34300.31807	31816.69798	0.767318551	0.459866656
mmu-miR-3082-3p	15.52606085	16.52887033	0.966052668	0.462397813
mmu-miR-467e-5p	75.82494836	72.41219382	0.809316438	0.463620667
mmu-miR-330-5p	146.233829	45.65116567	-0.803797103	0.464201203
mmu-miR-188-5p	11.91534903	12.98696954	1	0.469169411
mmu-miR-467d-5p	211.9487842	195.5916322	0.759885348	0.473809573
mmu-miR-125b-2-3p	0	0.787089063	2	0.474079813
mmu-miR-7036b-5p	0	0.787089063	2	0.474079813
mmu-miR-7046-3p	0	0.787089063	2	0.474079813
mmu-miR-7227-3p	0	0.787089063	2	0.474079813
mmu-miR-3110-3p	1.444284731	0	-2	0.474079813
mmu-miR-5626-5p	1.444284731	0	-2	0.474079813
mmu-miR-6971-3p	1.444284731	0	-2	0.474079813
mmu-miR-7235-3p	1.444284731	0	-2	0.474079813
mmu-miR-191-3p	72.9363789	22.4320383	-0.825321469	0.47483228
mmu-miR-205-5p	3.249640644	0.393544532	-2.169925001	0.475697832
mmu-miR-8105	3.249640644	0.393544532	-2.169925001	0.475697832
mmu-miR-139-3p	0.722142365	1.770950392	2.169925001	0.475697832
mmu-miR-1306-3p	17.33141677	4.525762114	-1.061400545	0.477516935
mmu-miR-466i-5p	8.304637201	1.770950392	-1.353636955	0.479372935
mmu-miR-7a-5p	266.831604	86.38302469	-0.751353425	0.481758508
mmu-miR-146a-5p	6651.653327	2196.765576	-0.722575668	0.485374414
mmu-miR-342-3p	2238.280261	2008.651289	0.719592825	0.487505099
mmu-miR-5104	29.2467658	8.46120743	-0.913585248	0.487514954
mmu-let-7a-1-3p	113.7374225	103.3054396	0.736965594	0.494708967
mmu-let-7c-2-3p	113.7374225	103.3054396	0.736965594	0.494708967
mmu-miR-700-5p	19.85891505	19.67722658	0.862496476	0.49479307
mmu-miR-7033-5p	18.05355913	4.919306645	-1	0.496211955
mmu-miR-5119	0.361071183	1.180633595	2.584962501	0.50573011
mmu-miR-1968-5p	2.166427096	0.196772266	-2.584962501	0.50573011
mmu-miR-672-5p	2.166427096	0.196772266	-2.584962501	0.50573011
mmu-miR-6932-5p	2.166427096	0.196772266	-2.584962501	0.50573011
mmu-miR-16-1-3p	67.15923997	60.9994024	0.736965594	0.506183291
mmu-miR-374b-5p	87.74029739	78.51213406	0.715432433	0.51188407

mmu-miR-532-3p	22.74748451	21.64494924	0.80407979	0.516045927
mmu-miR-200c-3p	32.13533526	9.838613291	-0.831877241	0.516947899
mmu-miR-210-3p	471.1978934	160.3693966	-0.679177842	0.517189746
mmu-miR-6996-5p	2.166427096	3.148356253	1.415037499	0.518624784
mmu-miR-128-1-5p	177.2859507	59.81876881	-0.691651701	0.521225638
mmu-miR-30b-3p	32.49640644	29.9093844	0.756074417	0.522291099
mmu-miR-136-3p	5.054996557	0.983861329	-1.485426827	0.523079432
mmu-miR-7042-5p	1.805355913	2.754811721	1.485426827	0.523079432
mmu-miR-187-3p	0.722142365	1.574178127	2	0.523087033
mmu-let-7g-3p	27.0803387	25.18685002	0.77118131	0.523415084
mmu-miR-5114	10.10999311	2.558039456	-1.106915204	0.525878457
mmu-miR-7082-3p	4.332854192	0.787089063	-1.584962501	0.526442254
mmu-miR-148b-3p	2273.304166	1947.455115	0.652555738	0.526916026
mmu-miR-21b	1.083213548	1.967722658	1.736965594	0.52742241
mmu-miR-6916-5p	1.083213548	1.967722658	1.736965594	0.52742241
mmu-miR-6989-3p	1.083213548	1.967722658	1.736965594	0.52742241
mmu-miR-6957-5p	3.249640644	4.132217582	1.222392421	0.529361322
mmu-miR-1934-5p	22.38641332	6.690257038	-0.866733469	0.529960818
mmu-miR-93-3p	54.52174858	17.90627619	-0.730610099	0.53576342
mmu-miR-30e-5p	12601.0232	10668.59871	0.635586198	0.536476816
mmu-miR-27a-5p	79.43566018	69.0670653	0.673967507	0.538078607
mmu-miR-101b-5p	1.083213548	0	-1.584962501	0.54232359
mmu-miR-299a-3p	1.083213548	0	-1.584962501	0.54232359
mmu-miR-3094-5p	1.083213548	0	-1.584962501	0.54232359
mmu-miR-379-3p	1.083213548	0	-1.584962501	0.54232359
mmu-miR-380-3p	1.083213548	0	-1.584962501	0.54232359
mmu-miR-431-5p	1.083213548	0	-1.584962501	0.54232359
mmu-miR-485-3p	1.083213548	0	-1.584962501	0.54232359
mmu-miR-543-3p	1.083213548	0	-1.584962501	0.54232359
mmu-miR-6238	1.083213548	0	-1.584962501	0.54232359
mmu-miR-6900-5p	1.083213548	0	-1.584962501	0.54232359
mmu-miR-6904-3p	1.083213548	0	-1.584962501	0.54232359
mmu-miR-6962-5p	1.083213548	0	-1.584962501	0.54232359
mmu-miR-6999-3p	1.083213548	0	-1.584962501	0.54232359
mmu-miR-7085-3p	1.083213548	0	-1.584962501	0.54232359
mmu-miR-7651-5p	1.083213548	0	-1.584962501	0.54232359
mmu-miR-7664-3p	1.083213548	0	-1.584962501	0.54232359
mmu-miR-7672-3p	1.083213548	0	-1.584962501	0.54232359
mmu-miR-1936	0	0.590316797	1.584962501	0.54232359

mmu-miR-1953	0	0.590316797	1.584962501	0.54232359
mmu-miR-1966-3p	0	0.590316797	1.584962501	0.54232359
mmu-miR-34b-3p	0	0.590316797	1.584962501	0.54232359
mmu-miR-465c-5p	0	0.590316797	1.584962501	0.54232359
mmu-miR-5624-5p	0	0.590316797	1.584962501	0.54232359
mmu-miR-592-5p	0	0.590316797	1.584962501	0.54232359
mmu-miR-6961-5p	0	0.590316797	1.584962501	0.54232359
mmu-miR-701-3p	0	0.590316797	1.584962501	0.54232359
mmu-miR-871-3p	0	0.590316797	1.584962501	0.54232359
mmu-miR-18a-5p	351.6833319	124.1632997	-0.626281767	0.551022903
mmu-miR-466g	93.87850749	32.27065159	-0.664815808	0.551111637
mmu-miR-429-3p	27.44140988	24.59653323	0.717856771	0.551807255
mmu-miR-466k	10.83213548	2.951583987	-1	0.553491276
mmu-miR-466h-3p	33.2185488	10.82247462	-0.742202243	0.557501222
mmu-miR-142b	1.805355913	2.558039456	1.378511623	0.559594206
mmu-miR-7237-3p	1.805355913	2.558039456	1.378511623	0.559594206
mmu-miR-466h-5p	37.91247418	12.59342501	-0.714245518	0.563362378
mmu-miR-1668	2.888569461	3.541900785	1.169925001	0.566065843
mmu-let-7f-1-3p	81.60208728	68.4767485	0.622764533	0.568032714
mmu-miR-152-5p	1.805355913	0.196772266	-2.321928095	0.568604695
mmu-miR-467b-3p	1.805355913	0.196772266	-2.321928095	0.568604695
mmu-miR-491-3p	1.805355913	0.196772266	-2.321928095	0.568604695
mmu-miR-7019-3p	1.805355913	0.196772266	-2.321928095	0.568604695
mmu-miR-652-5p	0.361071183	0.983861329	2.321928095	0.568604695
mmu-miR-351-5p	175.1195236	144.6276154	0.599759503	0.57001205
mmu-miR-6546-5p	3.249640644	0.590316797	-1.584962501	0.573736717
mmu-miR-21c	1.083213548	1.770950392	1.584962501	0.573736717
mmu-miR-3087-3p	1.083213548	1.770950392	1.584962501	0.573736717
mmu-miR-6418-3p	0.722142365	1.377405861	1.807354922	0.57661162
mmu-miR-183-5p	594.6842378	217.2365815	-0.577100383	0.578020825
mmu-miR-466d-5p	25.63605397	8.46120743	-0.723482365	0.584490699
mmu-miR-466n-5p	25.63605397	8.46120743	-0.723482365	0.584490699
mmu-miR-669d-5p	14.08177612	12.79019728	0.736965594	0.584668577
mmu-miR-301a-3p	2430.731202	902.397611	-0.553798822	0.589200895
mmu-miR-363-3p	1321.520529	496.0628821	-0.53784753	0.600602365
mmu-miR-425-3p	46.57818256	16.52887033	-0.618909833	0.603272752
mmu-miR-3470a	33.94069117	11.80633595	-0.647698256	0.604273557
mmu-miR-3091-5p	5.054996557	5.116078911	0.893084796	0.608250176
mmu-miR-107-3p	711.3102298	557.6526013	0.524644129	0.609901665

mmu-miR-340-5p	1142.790293	894.5267204	0.522392185	0.610357635
mmu-miR-744-5p	2476.226171	1934.468145	0.519550269	0.611321037
mmu-miR-25-5p	394.2897315	149.1533775	-0.526703103	0.612847478
mmu-miR-712-5p	15.16498967	4.919306645	-0.748461233	0.616231399
mmu-miR-9-5p	11.91534903	3.73867305	-0.796466606	0.619373863
mmu-miR-8096	6.138210105	5.903167974	0.819427754	0.620126829
mmu-miR-466a-5p	5.41606774	5.312851177	0.847996907	0.620544949
mmu-miR-669e-5p	2.888569461	0.590316797	-1.415037499	0.625216949
mmu-miR-676-3p	2.888569461	0.590316797	-1.415037499	0.625216949
mmu-miR-1931	4.693925375	1.180633595	-1.115477217	0.62569201
mmu-miR-1247-3p	0.722142365	0	-1	0.62622148
mmu-miR-193a-5p	0.722142365	0	-1	0.62622148
mmu-miR-1970	0.722142365	0	-1	0.62622148
mmu-miR-205-3p	0.722142365	0	-1	0.62622148
mmu-miR-3064-3p	0.722142365	0	-1	0.62622148
mmu-miR-3102-3p.2-3	0.722142365	0	-1	0.62622148
mmu-miR-3102-5p.2-5	0.722142365	0	-1	0.62622148
mmu-miR-3572-5p	0.722142365	0	-1	0.62622148
mmu-miR-369-3p	0.722142365	0	-1	0.62622148
mmu-miR-379-5p	0.722142365	0	-1	0.62622148
mmu-miR-433-3p	0.722142365	0	-1	0.62622148
mmu-miR-466l-3p	0.722142365	0	-1	0.62622148
mmu-miR-485-5p	0.722142365	0	-1	0.62622148
mmu-miR-487b-3p	0.722142365	0	-1	0.62622148
mmu-miR-669h-3p	0.722142365	0	-1	0.62622148
mmu-miR-679-5p	0.722142365	0	-1	0.62622148
mmu-miR-6922-3p	0.722142365	0	-1	0.62622148
mmu-miR-6922-5p	0.722142365	0	-1	0.62622148
mmu-miR-6952-3p	0.722142365	0	-1	0.62622148
mmu-miR-6958-3p	0.722142365	0	-1	0.62622148
mmu-miR-6977-5p	0.722142365	0	-1	0.62622148
mmu-miR-6985-3p	0.722142365	0	-1	0.62622148
mmu-miR-6997-3p	0.722142365	0	-1	0.62622148
mmu-miR-701-5p	0.722142365	0	-1	0.62622148
mmu-miR-702-5p	0.722142365	0	-1	0.62622148
mmu-miR-7031-5p	0.722142365	0	-1	0.62622148
mmu-miR-7068-5p	0.722142365	0	-1	0.62622148
mmu-miR-7069-5p	0.722142365	0	-1	0.62622148
mmu-miR-7213-5p	0.722142365	0	-1	0.62622148

mmu-miR-7240-5p	0.722142365	0	-1	0.62622148
mmu-miR-758-3p	0.722142365	0	-1	0.62622148
mmu-miR-7648-5p	0.722142365	0	-1	0.62622148
mmu-miR-8120	0.722142365	0	-1	0.62622148
mmu-miR-122-5p	0	0.393544532	1	0.62622148
mmu-miR-126b-5p	0	0.393544532	1	0.62622148
mmu-miR-129b-5p	0	0.393544532	1	0.62622148
mmu-miR-145a-3p	0	0.393544532	1	0.62622148
mmu-miR-181a-2-3p	0	0.393544532	1	0.62622148
mmu-miR-1948-5p	0	0.393544532	1	0.62622148
mmu-miR-200c-5p	0	0.393544532	1	0.62622148
mmu-miR-219b-5p	0	0.393544532	1	0.62622148
mmu-miR-3084-5p	0	0.393544532	1	0.62622148
mmu-miR-3087-5p	0	0.393544532	1	0.62622148
mmu-miR-450a-2-3p	0	0.393544532	1	0.62622148
mmu-miR-450b-3p	0	0.393544532	1	0.62622148
mmu-miR-500-5p	0	0.393544532	1	0.62622148
mmu-miR-503-5p	0	0.393544532	1	0.62622148
mmu-miR-5133	0	0.393544532	1	0.62622148
mmu-miR-6380	0	0.393544532	1	0.62622148
mmu-miR-6967-3p	0	0.393544532	1	0.62622148
mmu-miR-6988-3p	0	0.393544532	1	0.62622148
mmu-miR-7026-3p	0	0.393544532	1	0.62622148
mmu-miR-7036a-5p	0	0.393544532	1	0.62622148
mmu-miR-7051-5p	0	0.393544532	1	0.62622148
mmu-miR-7053-3p	0	0.393544532	1	0.62622148
mmu-miR-7054-3p	0	0.393544532	1	0.62622148
mmu-miR-7054-5p	0	0.393544532	1	0.62622148
mmu-miR-7058-5p	0	0.393544532	1	0.62622148
mmu-miR-7067-3p	0	0.393544532	1	0.62622148
mmu-miR-7089-5p	0	0.393544532	1	0.62622148
mmu-miR-7659-5p	0	0.393544532	1	0.62622148
mmu-miR-7671-3p	0	0.393544532	1	0.62622148
mmu-miR-7671-5p	0	0.393544532	1	0.62622148
mmu-miR-7679-3p	0	0.393544532	1	0.62622148
mmu-miR-99a-5p	0	0.393544532	1	0.62622148
mmu-miR-6911-3p	2.888569461	3.148356253	1	0.631399902
mmu-let-7i-3p	44.77282665	35.61578011	0.545649577	0.632040531
mmu-miR-22-5p	5.054996557	4.919306645	0.836501268	0.633170729

mmu-miR-3103-3p	6.86035247	1.967722658	-0.925999419	0.633514048
mmu-miR-190b-3p	0.722142365	1.180633595	1.584962501	0.637355634
mmu-miR-195a-3p	0.722142365	1.180633595	1.584962501	0.637355634
mmu-miR-455-3p	2.166427096	0.393544532	-1.584962501	0.637355634
mmu-miR-673-5p	2.166427096	0.393544532	-1.584962501	0.637355634
mmu-miR-20b-5p	311.6044306	120.8181712	-0.491121904	0.638024432
mmu-miR-877-5p	61.38210105	48.01243286	0.521346401	0.63807565
mmu-miR-27b-5p	337.2404846	131.2471013	-0.485735789	0.640993457
mmu-miR-1947-5p	46.57818256	17.31595939	-0.551795637	0.641238197
mmu-miR-7670-3p	3.971783009	0.983861329	-1.137503524	0.642355754
mmu-miR-99b-3p	1.805355913	2.164494924	1.137503524	0.642355754
mmu-miR-1247-5p	1.444284731	0.196772266	-2	0.642740637
mmu-miR-1938	1.444284731	0.196772266	-2	0.642740637
mmu-miR-199a-5p	1.444284731	0.196772266	-2	0.642740637
mmu-miR-3112-3p	1.444284731	0.196772266	-2	0.642740637
mmu-miR-3472	1.444284731	0.196772266	-2	0.642740637
mmu-miR-501-5p	1.444284731	0.196772266	-2	0.642740637
mmu-miR-6970-3p	1.444284731	0.196772266	-2	0.642740637
mmu-miR-7058-3p	1.444284731	0.196772266	-2	0.642740637
mmu-miR-7658-5p	1.444284731	0.196772266	-2	0.642740637
mmu-miR-1964-5p	0.361071183	0.787089063	2	0.642740637
mmu-miR-5132-3p	0.361071183	0.787089063	2	0.642740637
mmu-miR-6902-5p	0.361071183	0.787089063	2	0.642740637
mmu-miR-7021-5p	0.361071183	0.787089063	2	0.642740637
mmu-miR-7219-3p	0.361071183	0.787089063	2	0.642740637
mmu-miR-7677-3p	0.361071183	0.787089063	2	0.642740637
mmu-miR-7068-3p	9.387850749	2.951583987	-0.793549123	0.644992183
mmu-miR-574-3p	6.499281288	5.903167974	0.736965594	0.652727337
mmu-miR-151-3p	1815.826978	1359.696357	0.458414157	0.653051059
mmu-let-7j	4751.335693	3547.213636	0.45410947	0.655401499
mmu-miR-103-3p	3981.893002	2968.30963	0.451943524	0.656978166
mmu-let-7k	0	0.196772266	-1.60171E-16	0.657607203
mmu-miR-103-1-5p	0	0.196772266	-1.60171E-16	0.657607203
mmu-miR-1188-3p	0	0.196772266	-1.60171E-16	0.657607203
mmu-miR-1193-5p	0.361071183	0	-1.60171E-16	0.657607203
mmu-miR-124-5p	0.361071183	0	-1.60171E-16	0.657607203
mmu-miR-125b-1-3p	0.361071183	0	-1.60171E-16	0.657607203
mmu-miR-126b-3p	0	0.196772266	-1.60171E-16	0.657607203
mmu-miR-127-5p	0.361071183	0	-1.60171E-16	0.657607203

mmu-miR-129-1-3p	0.361071183	0	-1.60171E-16	0.657607203
mmu-miR-133a-3p	0	0.196772266	-1.60171E-16	0.657607203
mmu-miR-149-3p	0	0.196772266	-1.60171E-16	0.657607203
mmu-miR-1892	0.361071183	0	-1.60171E-16	0.657607203
mmu-miR-1951	0.361071183	0	-1.60171E-16	0.657607203
mmu-miR-1969	0.361071183	0	-1.60171E-16	0.657607203
mmu-miR-28b	0.361071183	0	-1.60171E-16	0.657607203
mmu-miR-299a-5p	0.361071183	0	-1.60171E-16	0.657607203
mmu-miR-3058-5p	0	0.196772266	-1.60171E-16	0.657607203
mmu-miR-3075-5p	0.361071183	0	-1.60171E-16	0.657607203
mmu-miR-3080-3p	0	0.196772266	-1.60171E-16	0.657607203
mmu-miR-3092-3p	0	0.196772266	-1.60171E-16	0.657607203
mmu-miR-3094-3p	0.361071183	0	-1.60171E-16	0.657607203
mmu-miR-3097-3p	0	0.196772266	-1.60171E-16	0.657607203
mmu-miR-3101-3p	0	0.196772266	-1.60171E-16	0.657607203
mmu-miR-3108-5p	0.361071183	0	-1.60171E-16	0.657607203
mmu-miR-3109-5p	0	0.196772266	-1.60171E-16	0.657607203
mmu-miR-323-3p	0.361071183	0	-1.60171E-16	0.657607203
mmu-miR-326-5p	0.361071183	0	-1.60171E-16	0.657607203
mmu-miR-338-5p	0	0.196772266	-1.60171E-16	0.657607203
mmu-miR-346-3p	0.361071183	0	-1.60171E-16	0.657607203
mmu-miR-351-3p	0	0.196772266	-1.60171E-16	0.657607203
mmu-miR-3618-5p	0.361071183	0	-1.60171E-16	0.657607203
mmu-miR-374c-3p	0	0.196772266	-1.60171E-16	0.657607203
mmu-miR-3966	0.361071183	0	-1.60171E-16	0.657607203
mmu-miR-431-3p	0.361071183	0	-1.60171E-16	0.657607203
mmu-miR-433-5p	0.361071183	0	-1.60171E-16	0.657607203
mmu-miR-465a-3p	0	0.196772266	-1.60171E-16	0.657607203
mmu-miR-465b-3p	0	0.196772266	-1.60171E-16	0.657607203
mmu-miR-465c-3p	0	0.196772266	-1.60171E-16	0.657607203
mmu-miR-470-5p	0	0.196772266	-1.60171E-16	0.657607203
mmu-miR-504-3p	0	0.196772266	-1.60171E-16	0.657607203
mmu-miR-5615-3p	0	0.196772266	-1.60171E-16	0.657607203
mmu-miR-5616-3p	0	0.196772266	-1.60171E-16	0.657607203
mmu-miR-5619-5p	0.361071183	0	-1.60171E-16	0.657607203
mmu-miR-5622-3p	0	0.196772266	-1.60171E-16	0.657607203
mmu-miR-5622-5p	0.361071183	0	-1.60171E-16	0.657607203
mmu-miR-6353	0	0.196772266	-1.60171E-16	0.657607203
mmu-miR-6516-3p	0	0.196772266	-1.60171E-16	0.657607203

mmu-miR-653-5p	0	0.196772266	-1.60171E-16	0.657607203
mmu-miR-6537-5p	0.361071183	0	-1.60171E-16	0.657607203
mmu-miR-6541	0	0.196772266	-1.60171E-16	0.657607203
mmu-miR-665-3p	0.361071183	0	-1.60171E-16	0.657607203
mmu-miR-667-5p	0.361071183	0	-1.60171E-16	0.657607203
mmu-miR-669m-3p	0.361071183	0	-1.60171E-16	0.657607203
mmu-miR-673-3p	0.361071183	0	-1.60171E-16	0.657607203
mmu-miR-676-5p	0	0.196772266	-1.60171E-16	0.657607203
mmu-miR-682	0.361071183	0	-1.60171E-16	0.657607203
mmu-miR-686	0.361071183	0	-1.60171E-16	0.657607203
mmu-miR-6898-5p	0.361071183	0	-1.60171E-16	0.657607203
mmu-miR-6900-3p	0.361071183	0	-1.60171E-16	0.657607203
mmu-miR-6908-5p	0	0.196772266	-1.60171E-16	0.657607203
mmu-miR-6910-3p	0	0.196772266	-1.60171E-16	0.657607203
mmu-miR-6910-5p	0.361071183	0	-1.60171E-16	0.657607203
mmu-miR-6914-3p	0.361071183	0	-1.60171E-16	0.657607203
mmu-miR-6915-3p	0.361071183	0	-1.60171E-16	0.657607203
mmu-miR-6918-3p	0	0.196772266	-1.60171E-16	0.657607203
mmu-miR-692	0.361071183	0	-1.60171E-16	0.657607203
mmu-miR-6924-3p	0	0.196772266	-1.60171E-16	0.657607203
mmu-miR-6924-5p	0	0.196772266	-1.60171E-16	0.657607203
mmu-miR-6925-5p	0	0.196772266	-1.60171E-16	0.657607203
mmu-miR-6926-3p	0	0.196772266	-1.60171E-16	0.657607203
mmu-miR-6937-3p	0	0.196772266	-1.60171E-16	0.657607203
mmu-miR-6938-3p	0	0.196772266	-1.60171E-16	0.657607203
mmu-miR-6942-5p	0.361071183	0	-1.60171E-16	0.657607203
mmu-miR-6943-5p	0	0.196772266	-1.60171E-16	0.657607203
mmu-miR-6944-3p	0	0.196772266	-1.60171E-16	0.657607203
mmu-miR-6946-3p	0	0.196772266	-1.60171E-16	0.657607203
mmu-miR-6947-3p	0.361071183	0	-1.60171E-16	0.657607203
mmu-miR-6955-3p	0	0.196772266	-1.60171E-16	0.657607203
mmu-miR-6956-3p	0.361071183	0	-1.60171E-16	0.657607203
mmu-miR-6959-5p	0	0.196772266	-1.60171E-16	0.657607203
mmu-miR-6962-3p	0.361071183	0	-1.60171E-16	0.657607203
mmu-miR-6964-3p	0	0.196772266	-1.60171E-16	0.657607203
mmu-miR-6965-3p	0	0.196772266	-1.60171E-16	0.657607203
mmu-miR-6974-3p	0.361071183	0	-1.60171E-16	0.657607203
mmu-miR-6975-3p	0.361071183	0	-1.60171E-16	0.657607203
mmu-miR-6979-3p	0	0.196772266	-1.60171E-16	0.657607203

mmu-miR-6984-3p	0.361071183	0	-1.60171E-16	0.657607203
mmu-miR-6987-5p	0.361071183	0	-1.60171E-16	0.657607203
mmu-miR-6994-3p	0.361071183	0	-1.60171E-16	0.657607203
mmu-miR-6999-5p	0.361071183	0	-1.60171E-16	0.657607203
mmu-miR-7004-5p	0	0.196772266	-1.60171E-16	0.657607203
mmu-miR-7010-5p	0.361071183	0	-1.60171E-16	0.657607203
mmu-miR-7014-5p	0	0.196772266	-1.60171E-16	0.657607203
mmu-miR-7023-3p	0	0.196772266	-1.60171E-16	0.657607203
mmu-miR-7028-5p	0.361071183	0	-1.60171E-16	0.657607203
mmu-miR-7029-5p	0.361071183	0	-1.60171E-16	0.657607203
mmu-miR-7030-3p	0	0.196772266	-1.60171E-16	0.657607203
mmu-miR-7031-3p	0	0.196772266	-1.60171E-16	0.657607203
mmu-miR-7035-3p	0	0.196772266	-1.60171E-16	0.657607203
mmu-miR-7036a-3p	0	0.196772266	-1.60171E-16	0.657607203
mmu-miR-7036b-3p	0.361071183	0	-1.60171E-16	0.657607203
mmu-miR-7037-5p	0	0.196772266	-1.60171E-16	0.657607203
mmu-miR-7038-3p	0.361071183	0	-1.60171E-16	0.657607203
mmu-miR-7038-5p	0.361071183	0	-1.60171E-16	0.657607203
mmu-miR-7039-3p	0.361071183	0	-1.60171E-16	0.657607203
mmu-miR-7039-5p	0	0.196772266	-1.60171E-16	0.657607203
mmu-miR-7042-3p	0	0.196772266	-1.60171E-16	0.657607203
mmu-miR-7045-5p	0.361071183	0	-1.60171E-16	0.657607203
mmu-miR-7048-3p	0.361071183	0	-1.60171E-16	0.657607203
mmu-miR-7048-5p	0	0.196772266	-1.60171E-16	0.657607203
mmu-miR-7060-5p	0.361071183	0	-1.60171E-16	0.657607203
mmu-miR-7061-5p	0	0.196772266	-1.60171E-16	0.657607203
mmu-miR-7072-3p	0.361071183	0	-1.60171E-16	0.657607203
mmu-miR-7072-5p	0	0.196772266	-1.60171E-16	0.657607203
mmu-miR-7079-5p	0.361071183	0	-1.60171E-16	0.657607203
mmu-miR-7084-3p	0.361071183	0	-1.60171E-16	0.657607203
mmu-miR-7092-5p	0	0.196772266	-1.60171E-16	0.657607203
mmu-miR-7093-5p	0.361071183	0	-1.60171E-16	0.657607203
mmu-miR-7116-3p	0	0.196772266	-1.60171E-16	0.657607203
mmu-miR-7646-3p	0.361071183	0	-1.60171E-16	0.657607203
mmu-miR-7649-3p	0	0.196772266	-1.60171E-16	0.657607203
mmu-miR-7654-3p	0	0.196772266	-1.60171E-16	0.657607203
mmu-miR-7656-5p	0.361071183	0	-1.60171E-16	0.657607203
mmu-miR-7658-3p	0	0.196772266	-1.60171E-16	0.657607203
mmu-miR-7663-3p	0	0.196772266	-1.60171E-16	0.657607203

mmu-miR-7669-3p	0.361071183	0	-1.60171E-16	0.657607203
mmu-miR-7669-5p	0	0.196772266	-1.60171E-16	0.657607203
mmu-miR-7675-5p	0.361071183	0	-1.60171E-16	0.657607203
mmu-miR-7676-3p	0.361071183	0	-1.60171E-16	0.657607203
mmu-miR-7679-5p	0.361071183	0	-1.60171E-16	0.657607203
mmu-miR-7685-5p	0.361071183	0	-1.60171E-16	0.657607203
mmu-miR-8092	0	0.196772266	-1.60171E-16	0.657607203
mmu-miR-8093	0.361071183	0	-1.60171E-16	0.657607203
mmu-miR-8098	0	0.196772266	-1.60171E-16	0.657607203
mmu-miR-8113	0.361071183	0	-1.60171E-16	0.657607203
mmu-miR-879-3p	0.361071183	0	-1.60171E-16	0.657607203
mmu-miR-5122	5.054996557	4.72253438	0.777607579	0.659292233
mmu-miR-301a-5p	21.30319978	7.674118367	-0.59724083	0.659838432
mmu-miR-455-5p	7.582494836	2.36126719	-0.807354922	0.662529333
mmu-miR-6973a-3p	13.35963376	11.01924689	0.597901556	0.663103368
mmu-miR-222-5p	21.66427096	7.870890633	-0.584962501	0.664905552
mmu-miR-542-5p	2.166427096	2.36126719	1	0.667171545
mmu-miR-5615-5p	2.166427096	2.36126719	1	0.667171545
mmu-miR-27b-3p	62631.04627	46159.23166	0.435497619	0.668017503
mmu-miR-98-3p	194.2562963	77.33150047	-0.45307686	0.668052671
mmu-miR-32-3p	22.02534214	8.067662898	-0.573185333	0.669820213
mmu-miR-7646-5p	7.221423653	6.296712506	0.678071905	0.671028401
mmu-miR-15b-5p	1581.49178	1160.759596	0.429539539	0.673299248
mmu-miR-30c-5p	3540.302946	2596.60682	0.428511147	0.673399473
mmu-miR-130b-3p	2085.908222	850.4497329	-0.418621422	0.680997973
mmu-miR-3058-3p	1.083213548	1.377405861	1.222392421	0.682683622
mmu-miR-335-5p	1.083213548	1.377405861	1.222392421	0.682683622
mmu-miR-126a-5p	215.9205672	87.36688602	-0.429585808	0.682777578
mmu-miR-92a-1-5p	3302.718108	1352.612555	-0.412148799	0.685162028
mmu-miR-5121	109.4045683	80.47985672	0.432783049	0.685207881
mmu-miR-193a-3p	8.304637201	2.754811721	-0.716207034	0.686684676
mmu-miR-500-3p	24.9139116	19.08690978	0.491388385	0.688702284
mmu-miR-19b-3p	2197.118146	1587.558641	0.406953821	0.688939894
mmu-miR-1258-5p	3.610711827	0.983861329	-1	0.689246012
mmu-miR-1895	3.610711827	0.983861329	-1	0.689246012
mmu-miR-1927	7.221423653	2.36126719	-0.736965594	0.692771954
mmu-miR-466b-5p	32.85747762	12.79019728	-0.485426827	0.69507847
mmu-miR-466o-5p	32.85747762	12.79019728	-0.485426827	0.69507847
mmu-miR-297a-5p	18.41463032	6.887029304	-0.543142325	0.696816223

mmu-miR-872-3p	74.01959244	54.1123731	0.423807709	0.697841753
mmu-miR-7a-1-3p	100.0167176	72.41219382	0.40981979	0.702202473
mmu-miR-297b-5p	15.88713204	5.903167974	-0.552541023	0.702291867
mmu-miR-421-3p	854.2944182	356.1578011	-0.386460376	0.705372281
mmu-miR-92a-2-5p	5.054996557	1.574178127	-0.807354922	0.705861307
mmu-miR-3068-3p	219.8923502	157.2210404	0.391753275	0.70643567
mmu-miR-125b-5p	0.722142365	0.983861329	1.321928095	0.706925564
mmu-miR-669h-5p	0.722142365	0.983861329	1.321928095	0.706925564
mmu-miR-6955-5p	0.722142365	0.983861329	1.321928095	0.706925564
mmu-miR-7212-3p	0.722142365	0.983861329	1.321928095	0.706925564
mmu-miR-9-3p	1.805355913	0.393544532	-1.321928095	0.706925564
mmu-miR-101b-3p	3612.156111	2561.778129	0.380041676	0.707919839
mmu-miR-3074-1-3p	23.83069806	9.248296493	-0.489805268	0.708993587
mmu-miR-466f-3p	36.10711827	14.3643754	-0.454031631	0.709104546
mmu-miR-874-5p	2.166427096	2.164494924	0.874469118	0.711953858
mmu-miR-146b-5p	738.0294973	309.7195464	-0.376959655	0.712550932
mmu-miR-1929-5p	1.444284731	1.574178127	1	0.715331921
mmu-miR-5126	1.444284731	1.574178127	1	0.715331921
mmu-miR-6412	1.444284731	1.574178127	1	0.715331921
mmu-miR-6539	64.27067051	26.36748362	-0.409644241	0.716991198
mmu-miR-17-5p	2100.35107	890.3945028	-0.362357512	0.721259493
mmu-miR-6960-5p	3.249640644	2.951583987	0.736965594	0.721381498
mmu-miR-345-5p	71.85316535	29.71261214	-0.398219881	0.721604798
mmu-miR-324-3p	80.51887373	33.45128519	-0.391508964	0.72344849
mmu-miR-21a-3p	2521.72114	1072.408849	-0.357797329	0.72441026
mmu-miR-324-5p	35.02390472	14.16760314	-0.429987841	0.724578577
mmu-miR-190b-5p	15.16498967	11.41279142	0.465663572	0.729134503
mmu-miR-1964-3p	178.3691642	124.7536165	0.359971799	0.730551987
mmu-miR-5123	4.332854192	1.377405861	-0.777607579	0.73085754
mmu-miR-106a-3p	1.083213548	0.196772266	-1.584962501	0.732212233
mmu-miR-125a-3p	1.083213548	0.196772266	-1.584962501	0.732212233
mmu-miR-184-3p	0.361071183	0.590316797	1.584962501	0.732212233
mmu-miR-190a-5p	1.083213548	0.196772266	-1.584962501	0.732212233
mmu-miR-193b-3p	0.361071183	0.590316797	1.584962501	0.732212233
mmu-miR-218-5p	1.083213548	0.196772266	-1.584962501	0.732212233
mmu-miR-29b-2-5p	0.361071183	0.590316797	1.584962501	0.732212233
mmu-miR-3102-3p	1.083213548	0.196772266	-1.584962501	0.732212233
mmu-miR-449b	0.361071183	0.590316797	1.584962501	0.732212233
mmu-miR-5100	1.083213548	0.196772266	-1.584962501	0.732212233

mmu-miR-5106	1.083213548	0.196772266	-1.584962501	0.732212233
mmu-miR-5620-3p	0.361071183	0.590316797	1.584962501	0.732212233
mmu-miR-669d-2-3p	1.083213548	0.196772266	-1.584962501	0.732212233
mmu-miR-669l-3p	1.083213548	0.196772266	-1.584962501	0.732212233
mmu-miR-6933-5p	1.083213548	0.196772266	-1.584962501	0.732212233
mmu-miR-6952-5p	1.083213548	0.196772266	-1.584962501	0.732212233
mmu-miR-6967-5p	1.083213548	0.196772266	-1.584962501	0.732212233
mmu-miR-6990-3p	1.083213548	0.196772266	-1.584962501	0.732212233
mmu-miR-7073-5p	1.083213548	0.196772266	-1.584962501	0.732212233
mmu-miR-7091-5p	1.083213548	0.196772266	-1.584962501	0.732212233
mmu-miR-7117-5p	0.361071183	0.590316797	1.584962501	0.732212233
mmu-miR-7655-3p	1.083213548	0.196772266	-1.584962501	0.732212233
mmu-miR-7b-5p	0.361071183	0.590316797	1.584962501	0.732212233
mmu-miR-1195	14.80391849	5.706395709	-0.499571009	0.732858533
mmu-miR-221-5p	10.10999311	3.73867305	-0.559427409	0.732869591
mmu-miR-1258-3p	5.777138922	1.967722658	-0.678071905	0.735047501
mmu-miR-24-3p	753.5555582	520.2658708	0.341291576	0.737689153
mmu-miR-690	20.21998623	8.067662898	-0.449802917	0.739542569
mmu-miR-8112	31.05212171	22.23526604	0.393914208	0.740680316
mmu-miR-33-5p	93.87850749	65.13161998	0.348319594	0.745765874
mmu-miR-1191b-5p	2.888569461	2.558039456	0.700439718	0.746928896
mmu-miR-3064-5p	1.083213548	1.180633595	1	0.747068423
mmu-miR-6943-3p	1.083213548	1.180633595	1	0.747068423
mmu-miR-6974-5p	1.083213548	1.180633595	1	0.747068423
mmu-miR-107-5p	2.166427096	0.590316797	-1	0.747068423
mmu-miR-1930-3p	2.166427096	0.590316797	-1	0.747068423
mmu-miR-702-3p	2.166427096	0.590316797	-1	0.747068423
mmu-miR-704	2.166427096	0.590316797	-1	0.747068423
mmu-miR-183-3p	11.19320666	4.328989848	-0.494764692	0.754030088
mmu-miR-17-3p	376.5972435	164.1080697	-0.322619869	0.754227772
mmu-miR-3963	45.8560402	19.48045432	-0.359328067	0.758597222
mmu-miR-188-3p	3.610711827	1.180633595	-0.736965594	0.760397537
mmu-miR-1930-5p	3.610711827	1.180633595	-0.736965594	0.760397537
mmu-miR-3473f	2.166427096	1.967722658	0.736965594	0.760397537
mmu-miR-8118	2.166427096	1.967722658	0.736965594	0.760397537
mmu-miR-3066-3p	5.054996557	1.770950392	-0.637429921	0.760821
mmu-miR-126a-3p	27.44140988	11.60956368	-0.365284464	0.772631112
mmu-miR-5107-5p	2.527498279	0.787089063	-0.807354922	0.77514265
mmu-miR-3066-5p	12.27642021	8.854751962	0.404390255	0.775275165

mmu-miR-467a-5p	256.3605397	171.3886435	0.294853694	0.77536593
mmu-miR-467b-5p	256.3605397	171.3886435	0.294853694	0.77536593
mmu-miR-297a-3p	20.94212859	14.56114767	0.351472371	0.781789191
mmu-miR-297b-3p	20.94212859	14.56114767	0.351472371	0.781789191
mmu-miR-297c-3p	20.94212859	14.56114767	0.351472371	0.781789191
mmu-miR-467c-3p	9.387850749	3.73867305	-0.452512205	0.784734743
mmu-miR-186-3p	7.582494836	2.951583987	-0.485426827	0.785171848
mmu-miR-425-5p	1615.793542	1065.128275	0.274540706	0.786245239
mmu-miR-3471	1.444284731	0.393544532	-1	0.787530168
mmu-miR-6966-3p	1.444284731	0.393544532	-1	0.787530168
mmu-miR-7017-5p	1.444284731	0.393544532	-1	0.787530168
mmu-miR-1950	0.722142365	0.787089063	1	0.787530168
mmu-miR-1956	0.722142365	0.787089063	1	0.787530168
mmu-miR-34b-5p	0.722142365	0.787089063	1	0.787530168
mmu-miR-503-3p	0.722142365	0.787089063	1	0.787530168
mmu-miR-5132-5p	0.722142365	0.787089063	1	0.787530168
mmu-miR-6994-5p	0.722142365	0.787089063	1	0.787530168
mmu-miR-5099	5356.852066	3524.388053	0.271743715	0.787795868
mmu-miR-669b-5p	42.24532837	18.49659299	-0.315775868	0.788669281
mmu-miR-467e-3p	11.55427784	4.72253438	-0.415037499	0.789038407
mmu-miR-423-3p	7939.955307	3591.290623	-0.268872091	0.78992187
mmu-miR-669k-5p	6.138210105	2.36126719	-0.502500341	0.793409024
mmu-miR-466c-5p	19.13677268	13.18374181	0.338168736	0.793414641
mmu-miR-669l-5p	26.35819633	17.90627619	0.317970081	0.794699774
mmu-miR-1933-3p	16.6092744	7.083801569	-0.353636955	0.800886901
mmu-miR-362-5p	7.943566018	5.706395709	0.398549376	0.802465461
mmu-miR-221-3p	67.15923997	44.27375981	0.27462238	0.802545612
mmu-miR-98-5p	2449.867974	1588.739274	0.250934771	0.803844311
mmu-miR-148b-5p	254.1941126	115.8988646	-0.257307795	0.804097922
mmu-miR-30c-1-3p	164.2873881	107.0441126	0.257740106	0.805340139
mmu-miR-1981-3p	37.19033181	16.52887033	-0.294183104	0.80590314
mmu-miR-19a-5p	37.551403	16.72564259	-0.291048782	0.807627567
mmu-miR-669f-3p	10.83213548	4.525762114	-0.38332864	0.807693174
mmu-miR-3060-3p	15.16498967	6.493484772	-0.347923303	0.808149276
mmu-miR-669a-5p	326.0472779	149.9404666	-0.24493499	0.812119105
mmu-miR-669p-5p	326.0472779	149.9404666	-0.24493499	0.812119105
mmu-miR-671-3p	527.5249979	339.8257031	0.241311905	0.812886974
mmu-miR-301b-5p	3.249640644	1.180633595	-0.584962501	0.812888621
mmu-miR-19b-1-5p	3.610711827	2.754811721	0.485426827	0.81320627

mmu-miR-1191a	44.41175547	29.12229534	0.26693886	0.815113025
mmu-miR-15b-3p	381.2911689	245.1782432	0.238694234	0.815682664
mmu-miR-23b-3p	795.8008866	510.6240298	0.235610315	0.816487726
mmu-miR-24-1-5p	5.41606774	3.935445316	0.415037499	0.817160824
mmu-miR-1934-3p	1.083213548	0.983861329	0.736965594	0.819814055
mmu-miR-1946a	1.083213548	0.983861329	0.736965594	0.819814055
mmu-miR-3112-5p	1.083213548	0.983861329	0.736965594	0.819814055
mmu-miR-19b-2-5p	1.805355913	0.590316797	-0.736965594	0.819814055
mmu-miR-7667-5p	1.805355913	0.590316797	-0.736965594	0.819814055
mmu-miR-331-3p	50.54996557	32.86096839	0.254421276	0.821305668
mmu-miR-3061-3p	38.63461654	17.51273166	-0.265733555	0.823076112
mmu-miR-3091-3p	7.582494836	3.148356253	-0.392317423	0.823755561
mmu-miR-331-5p	7.582494836	3.148356253	-0.392317423	0.823755561
mmu-miR-138-5p	31.77426407	14.3643754	-0.26960706	0.825684719
mmu-miR-195a-5p	12.27642021	5.312851177	-0.332575339	0.825913737
mmu-miR-7667-3p	12.27642021	5.312851177	-0.332575339	0.825913737
mmu-miR-6538	2.527498279	1.967722658	0.514573173	0.82645337
mmu-miR-872-5p	517.0539336	243.0137483	-0.213520451	0.834355782
mmu-miR-466q	1.444284731	1.180633595	0.584962501	0.841564838
mmu-miR-6935-5p	1.444284731	1.180633595	0.584962501	0.841564838
mmu-miR-6546-3p	2.166427096	0.787089063	-0.584962501	0.841564838
mmu-miR-7653-5p	2.166427096	0.787089063	-0.584962501	0.841564838
mmu-miR-185-3p	19.13677268	8.657979696	-0.268488836	0.842019567
mmu-miR-20a-5p	1299.134115	616.0939643	-0.200571678	0.842903001
mmu-miR-148a-5p	262.1376786	123.9665275	-0.20461772	0.843214375
mmu-miR-106b-5p	722.1423653	451.7891223	0.199122642	0.844508284
mmu-miR-192-3p	0.722142365	0.196772266	-1	0.844691302
mmu-miR-194-2-3p	0.722142365	0.196772266	-1	0.844691302
mmu-miR-26a-1-3p	0.722142365	0.196772266	-1	0.844691302
mmu-miR-298-3p	0.722142365	0.196772266	-1	0.844691302
mmu-miR-411-3p	0.722142365	0.196772266	-1	0.844691302
mmu-miR-467h	0.722142365	0.196772266	-1	0.844691302
mmu-miR-493-3p	0.722142365	0.196772266	-1	0.844691302
mmu-miR-669d-3p	0.722142365	0.196772266	-1	0.844691302
mmu-miR-6946-5p	0.722142365	0.196772266	-1	0.844691302
mmu-miR-6998-3p	0.722142365	0.196772266	-1	0.844691302
mmu-miR-7032-3p	0.722142365	0.196772266	-1	0.844691302
mmu-miR-7075-3p	0.722142365	0.196772266	-1	0.844691302
mmu-miR-7652-3p	0.722142365	0.196772266	-1	0.844691302

mmu-miR-10a-3p	0.361071183	0.393544532	1	0.844691302
mmu-miR-1199-3p	0.361071183	0.393544532	1	0.844691302
mmu-miR-1946b	0.361071183	0.393544532	1	0.844691302
mmu-miR-200b-5p	0.361071183	0.393544532	1	0.844691302
mmu-miR-3077-3p	0.361071183	0.393544532	1	0.844691302
mmu-miR-3962	0.361071183	0.393544532	1	0.844691302
mmu-miR-5624-3p	0.361071183	0.393544532	1	0.844691302
mmu-miR-668-3p	0.361071183	0.393544532	1	0.844691302
mmu-miR-6913-3p	0.361071183	0.393544532	1	0.844691302
mmu-miR-6927-3p	0.361071183	0.393544532	1	0.844691302
mmu-miR-6986-5p	0.361071183	0.393544532	1	0.844691302
mmu-miR-7670-5p	0.361071183	0.393544532	1	0.844691302
mmu-miR-423-5p	4166.761448	2600.14872	0.195424026	0.846239785
mmu-let-7d-3p	1797.051276	1116.289064	0.188835477	0.851640309
mmu-miR-677-5p	26.71926752	12.39665275	-0.232173442	0.853475136
mmu-miR-669o-5p	33.57961999	21.25140471	0.215728691	0.855316395
mmu-miR-92a-3p	161486.92	77561.72402	-0.18224415	0.856236282
mmu-miR-7081-3p	2.527498279	0.983861329	-0.485426827	0.8575271
mmu-miR-3057-5p	49.10568084	23.21912737	-0.204819792	0.858444522
mmu-miR-671-5p	30.69105053	19.28368205	0.205318908	0.864010671
mmu-miR-466e-5p	10.83213548	4.919306645	-0.263034406	0.865669493
mmu-miR-7648-3p	2.888569461	1.180633595	-0.415037499	0.869895543
mmu-miR-7687-5p	8.665708384	3.935445316	-0.263034406	0.874290828
mmu-miR-1981-5p	38.63461654	18.49659299	-0.186878135	0.874430437
mmu-miR-1960	10.10999311	6.493484772	0.237039197	0.875244072
mmu-miR-709	3.249640644	1.377405861	-0.362570079	0.879851024
mmu-miR-3572-3p	2.527498279	1.770950392	0.362570079	0.879851024
mmu-miR-146b-3p	1.083213548	0.393544532	-0.584962501	0.882871745
mmu-miR-3062-5p	1.083213548	0.393544532	-0.584962501	0.882871745
mmu-miR-3101-5p	1.083213548	0.393544532	-0.584962501	0.882871745
mmu-miR-328-5p	1.083213548	0.393544532	-0.584962501	0.882871745
mmu-miR-466m-3p	1.083213548	0.393544532	-0.584962501	0.882871745
mmu-miR-491-5p	1.083213548	0.393544532	-0.584962501	0.882871745
mmu-miR-181b-2-3p	0.722142365	0.590316797	0.584962501	0.882871745
mmu-miR-181d-3p	0.722142365	0.590316797	0.584962501	0.882871745
mmu-miR-219b-3p	0.722142365	0.590316797	0.584962501	0.882871745
mmu-miR-466j	0.722142365	0.590316797	0.584962501	0.882871745
mmu-miR-486b-3p	0.722142365	0.590316797	0.584962501	0.882871745
mmu-miR-5127	0.722142365	0.590316797	0.584962501	0.882871745

mmu-miR-5626-3p	0.722142365	0.590316797	0.584962501	0.882871745
mmu-miR-7063-5p	0.722142365	0.590316797	0.584962501	0.882871745
mmu-miR-7654-5p	0.722142365	0.590316797	0.584962501	0.882871745
mmu-miR-669a-3p	145.1506154	87.95720282	0.15307933	0.884077368
mmu-miR-669o-3p	145.1506154	87.95720282	0.15307933	0.884077368
mmu-miR-3098-5p	6.499281288	2.951583987	-0.263034406	0.885539062
mmu-miR-3076-3p	3.610711827	1.574178127	-0.321928095	0.888091248
mmu-miR-19a-3p	510.9157235	253.0491338	-0.13791141	0.892371859
mmu-miR-20b-3p	15.52606085	7.477346101	-0.178337241	0.898796707
mmu-miR-3079-3p	1.444284731	0.590316797	-0.415037499	0.902994223
mmu-miR-7071-3p	1.444284731	0.590316797	-0.415037499	0.902994223
mmu-miR-7661-3p	1.444284731	0.590316797	-0.415037499	0.902994223
mmu-miR-7668-5p	1.444284731	0.590316797	-0.415037499	0.902994223
mmu-miR-1943-3p	1.083213548	0.787089063	0.415037499	0.902994223
mmu-miR-219c-5p	1.083213548	0.787089063	0.415037499	0.902994223
mmu-miR-669b-3p	1.083213548	0.787089063	0.415037499	0.902994223
mmu-miR-693-3p	1.083213548	0.787089063	0.415037499	0.902994223
mmu-miR-7012-3p	1.083213548	0.787089063	0.415037499	0.902994223
mmu-miR-669c-5p	89.5456533	53.32528404	0.127952731	0.905419657
mmu-miR-1198-5p	243.0009059	143.8405263	0.119264901	0.907992985
mmu-miR-466f	5.054996557	2.36126719	-0.222392421	0.910902244
mmu-miR-466f-5p	5.054996557	2.36126719	-0.222392421	0.910902244
mmu-miR-6990-5p	1.805355913	0.787089063	-0.321928095	0.915881938
mmu-miR-23b-5p	1.444284731	0.983861329	0.321928095	0.915881938
mmu-miR-328-3p	283.8019496	143.4469818	-0.108610498	0.916009081
mmu-miR-8114	29.96890816	14.9546922	-0.127111918	0.917277029
mmu-miR-1949	5.054996557	3.148356253	0.192645078	0.918705681
mmu-miR-3084-3p	5.054996557	3.148356253	0.192645078	0.918705681
mmu-miR-340-3p	51.9942503	26.17071135	-0.114642566	0.919598949
mmu-miR-2137	2.166427096	0.983861329	-0.263034406	0.925051117
mmu-miR-3069-3p	2.166427096	0.983861329	-0.263034406	0.925051117
mmu-miR-669p-3p	2.166427096	0.983861329	-0.263034406	0.925051117
mmu-miR-146a-3p	1.805355913	1.180633595	0.263034406	0.925051117
mmu-miR-182-3p	1.805355913	1.180633595	0.263034406	0.925051117
mmu-miR-3470b	58.49353159	34.23837425	0.103093493	0.926250649
mmu-miR-3079-5p	2.527498279	1.180633595	-0.222392421	0.93200748
mmu-miR-7675-3p	2.527498279	1.180633595	-0.222392421	0.93200748
mmu-miR-185-5p	27.0803387	13.77405861	-0.099535674	0.936152655
mmu-miR-466m-5p	2.888569461	1.377405861	-0.192645078	0.93752104

mmu-miR-669m-5p	2.888569461	1.377405861	-0.192645078	0.93752104
mmu-miR-714	2.888569461	1.377405861	-0.192645078	0.93752104
mmu-miR-20a-3p	35.02390472	17.90627619	-0.092118202	0.938420425
mmu-miR-8094	2.888569461	1.770950392	0.169925001	0.942031789
mmu-miR-3068-5p	8.665708384	5.116078911	0.115477217	0.942342401
mmu-miR-141-5p	9.026779566	5.312851177	0.111031312	0.943909679
mmu-miR-497a-5p	9.026779566	5.312851177	0.111031312	0.943909679
mmu-miR-128-3p	1117.51531	579.8878674	-0.070692349	0.944231056
mmu-miR-1198-3p	3.610711827	1.770950392	-0.152003093	0.945811793
mmu-miR-3473g	3.610711827	1.770950392	-0.152003093	0.945811793
mmu-miR-669a-3-3p	3.249640644	1.967722658	0.152003093	0.945811793
mmu-miR-182-5p	7675.29013	3993.296362	-0.066884752	0.946942062
mmu-miR-219c-3p	3.971783009	1.967722658	-0.137503524	0.949039558
mmu-miR-148a-3p	6104.630485	3478.146571	0.064171147	0.949100109
mmu-miR-1955-5p	11.91534903	6.09994024	-0.090197809	0.951669328
mmu-miR-466i-3p	12.27642021	6.296712506	-0.087462841	0.952744754
mmu-miR-466n-3p	12.27642021	6.296712506	-0.087462841	0.952744754
mmu-miR-505-3p	4.332854192	2.558039456	0.115477217	0.954293802
mmu-miR-467c-5p	256.3605397	145.4147044	0.05775534	0.955304528
mmu-miR-8103	14.08177612	7.280573835	-0.075948853	0.9574394
mmu-miR-29b-1-5p	6.138210105	3.541900785	0.08246216	0.963226883
mmu-miR-106b-3p	1019.303949	573.0008381	0.044781227	0.964661158
mmu-miR-29c-5p	7.221423653	3.73867305	-0.074000581	0.965787255
mmu-miR-3061-5p	7.943566018	4.132217582	-0.067114196	0.967981278
mmu-miR-21a-5p	83546.4556	44305.0466	-0.039351196	0.968737094
mmu-miR-6948-3p	8.665708384	4.525762114	-0.061400545	0.969884549
mmu-miR-219a-1-3p	75.10280599	41.91249262	0.034269902	0.974966748
mmu-miR-1194	13.72070494	7.280573835	-0.038474148	0.978500915
mmu-miR-186-5p	10750.17232	5750.669468	-0.026801574	0.978715235
mmu-miR-669f-5p	15.16498967	8.067662898	-0.034765418	0.98008501
mmu-miR-467a-3p	88.46243975	47.42211606	-0.023748603	0.982467825
mmu-miR-467d-3p	88.46243975	47.42211606	-0.023748603	0.982467825
mmu-miR-466d-3p	46.57818256	24.99007776	-0.022542569	0.984294614
mmu-miR-3074-5p	40.07890127	22.03849377	0.012939056	0.991134947
mmu-miR-484	2361.044463	1293.777648	0.007920871	0.99372294
mmu-miR-501-3p	92.43422276	50.57047231	0.005624549	0.995832236
mmu-miR-5103	3.971783009	2.164494924	0	1
mmu-miR-5107-3p	4.693925375	2.558039456	-4.44089E-16	1
mmu-miR-5116	2.527498279	1.377405861	-4.44089E-16	1

mmu-miR-7059-5p	3.249640644	1.770950392	0	1
mmu-miR-1291	2.166427096	1.180633595	0	1
mmu-miR-466p-5p	18.05355913	9.838613291	0	1
mmu-miR-669e-3p	1.083213548	0.590316797	0	1
mmu-miR-6948-5p	1.083213548	0.590316797	0	1
mmu-miR-7012-5p	1.083213548	0.590316797	0	1
mmu-let-7a-2-3p	0	0	-1.60171E-16	1
mmu-let-7c-1-3p	0	0	-1.60171E-16	1
mmu-let-7e-3p	0.361071183	0.196772266	-1.60171E-16	1
mmu-miR-100-3p	0	0	-1.60171E-16	1
mmu-miR-100-5p	0	0	-1.60171E-16	1
mmu-miR-105	0	0	-1.60171E-16	1
mmu-miR-10b-3p	0	0	-1.60171E-16	1
mmu-miR-1188-5p	0	0	-1.60171E-16	1
mmu-miR-1190	0	0	-1.60171E-16	1
mmu-miR-1191b-3p	0	0	-1.60171E-16	1
mmu-miR-1192	0	0	-1.60171E-16	1
mmu-miR-1193-3p	0	0	-1.60171E-16	1
mmu-miR-1197-3p	0	0	-1.60171E-16	1
mmu-miR-1197-5p	0	0	-1.60171E-16	1
mmu-miR-122-3p	0	0	-1.60171E-16	1
mmu-miR-1224-3p	0	0	-1.60171E-16	1
mmu-miR-1224-5p	0	0	-1.60171E-16	1
mmu-miR-1231-3p	0	0	-1.60171E-16	1
mmu-miR-1231-5p	0	0	-1.60171E-16	1
mmu-miR-124-3p	0	0	-1.60171E-16	1
mmu-miR-1249-5p	0	0	-1.60171E-16	1
mmu-miR-1251-3p	0	0	-1.60171E-16	1
mmu-miR-1251-5p	0	0	-1.60171E-16	1
mmu-miR-1264-3p	0	0	-1.60171E-16	1
mmu-miR-1264-5p	0	0	-1.60171E-16	1
mmu-miR-128-2-5p	0	0	-1.60171E-16	1
mmu-miR-1298-3p	0	0	-1.60171E-16	1
mmu-miR-1298-5p	0	0	-1.60171E-16	1
mmu-miR-129b-3p	0	0	-1.60171E-16	1
mmu-miR-130a-5p	0	0	-1.60171E-16	1
mmu-miR-130c	0	0	-1.60171E-16	1
mmu-miR-133a-5p	0	0	-1.60171E-16	1
mmu-miR-133b-3p	0	0	-1.60171E-16	1

mmu-miR-133b-5p	0	0	-1.60171E-16	1
mmu-miR-133c	0	0	-1.60171E-16	1
mmu-miR-134-3p	0	0	-1.60171E-16	1
mmu-miR-135a-1-3p	0	0	-1.60171E-16	1
mmu-miR-135a-2-3p	0	0	-1.60171E-16	1
mmu-miR-135a-5p	0	0	-1.60171E-16	1
mmu-miR-135b-3p	0	0	-1.60171E-16	1
mmu-miR-135b-5p	0	0	-1.60171E-16	1
mmu-miR-136-5p	0	0	-1.60171E-16	1
mmu-miR-137-3p	0	0	-1.60171E-16	1
mmu-miR-137-5p	0	0	-1.60171E-16	1
mmu-miR-138-1-3p	0	0	-1.60171E-16	1
mmu-miR-138-2-3p	0.361071183	0.196772266	-1.60171E-16	1
mmu-miR-143-5p	0	0	-1.60171E-16	1
mmu-miR-145a-5p	0	0	-1.60171E-16	1
mmu-miR-145b	0	0	-1.60171E-16	1
mmu-miR-152-3p	0	0	-1.60171E-16	1
mmu-miR-153-3p	0	0	-1.60171E-16	1
mmu-miR-153-5p	0	0	-1.60171E-16	1
mmu-miR-154-3p	0	0	-1.60171E-16	1
mmu-miR-154-5p	0	0	-1.60171E-16	1
mmu-miR-184-5p	0	0	-1.60171E-16	1
mmu-miR-187-5p	0	0	-1.60171E-16	1
mmu-miR-1893	0	0	-1.60171E-16	1
mmu-miR-1894-3p	0	0	-1.60171E-16	1
mmu-miR-1894-5p	0	0	-1.60171E-16	1
mmu-miR-1896	0	0	-1.60171E-16	1
mmu-miR-1897-3p	0	0	-1.60171E-16	1
mmu-miR-1897-5p	0	0	-1.60171E-16	1
mmu-miR-1898	0	0	-1.60171E-16	1
mmu-miR-1899	0	0	-1.60171E-16	1
mmu-miR-1900	0	0	-1.60171E-16	1
mmu-miR-1901	0	0	-1.60171E-16	1
mmu-miR-1902	0	0	-1.60171E-16	1
mmu-miR-1903	0	0	-1.60171E-16	1
mmu-miR-1904	0	0	-1.60171E-16	1
mmu-miR-1905	0	0	-1.60171E-16	1
mmu-miR-1906	0	0	-1.60171E-16	1
mmu-miR-1907	0	0	-1.60171E-16	1

mmu-miR-190a-3p	0	0	-1.60171E-16	1
mmu-miR-1912-3p	0	0	-1.60171E-16	1
mmu-miR-1912-5p	0	0	-1.60171E-16	1
mmu-miR-1928	0	0	-1.60171E-16	1
mmu-miR-1929-3p	0	0	-1.60171E-16	1
mmu-miR-1932	0	0	-1.60171E-16	1
mmu-miR-1933-5p	0	0	-1.60171E-16	1
mmu-miR-193b-5p	0	0	-1.60171E-16	1
mmu-miR-194-1-3p	0	0	-1.60171E-16	1
mmu-miR-1941-3p	0	0	-1.60171E-16	1
mmu-miR-1941-5p	0	0	-1.60171E-16	1
mmu-miR-1942	0	0	-1.60171E-16	1
mmu-miR-1947-3p	0	0	-1.60171E-16	1
mmu-miR-1952	0	0	-1.60171E-16	1
mmu-miR-1954	0.361071183	0.196772266	-1.60171E-16	1
mmu-miR-1957b	0	0	-1.60171E-16	1
mmu-miR-1958	0.361071183	0.196772266	-1.60171E-16	1
mmu-miR-195b	0	0	-1.60171E-16	1
mmu-miR-1961	0	0	-1.60171E-16	1
mmu-miR-1962	0	0	-1.60171E-16	1
mmu-miR-1963	0	0	-1.60171E-16	1
mmu-miR-1966-5p	0	0	-1.60171E-16	1
mmu-miR-1967	0	0	-1.60171E-16	1
mmu-miR-1968-3p	0	0	-1.60171E-16	1
mmu-miR-196a-1-3p	0	0	-1.60171E-16	1
mmu-miR-196a-2-3p	0	0	-1.60171E-16	1
mmu-miR-196a-5p	0	0	-1.60171E-16	1
mmu-miR-196b-3p	0	0	-1.60171E-16	1
mmu-miR-1971	0	0	-1.60171E-16	1
mmu-miR-1982-3p	1.444284731	0.787089063	-2.22045E-16	1
mmu-miR-1982-5p	0	0	-1.60171E-16	1
mmu-miR-199b-5p	0	0	-1.60171E-16	1
mmu-miR-1a-1-5p	0	0	-1.60171E-16	1
mmu-miR-1a-2-5p	0	0	-1.60171E-16	1
mmu-miR-1a-3p	0	0	-1.60171E-16	1
mmu-miR-1b-3p	0.361071183	0.196772266	-1.60171E-16	1
mmu-miR-1b-5p	0	0	-1.60171E-16	1
mmu-miR-200a-5p	1.444284731	0.787089063	-2.22045E-16	1
mmu-miR-201-3p	0	0	-1.60171E-16	1

mmu-miR-201-5p	0	0	-1.60171E-16	1
mmu-miR-202-3p	0	0	-1.60171E-16	1
mmu-miR-202-5p	0	0	-1.60171E-16	1
mmu-miR-204-3p	0	0	-1.60171E-16	1
mmu-miR-204-5p	0.361071183	0.196772266	-1.60171E-16	1
mmu-miR-206-3p	0	0	-1.60171E-16	1
mmu-miR-206-5p	0	0	-1.60171E-16	1
mmu-miR-207	0	0	-1.60171E-16	1
mmu-miR-208a-3p	0	0	-1.60171E-16	1
mmu-miR-208a-5p	0	0	-1.60171E-16	1
mmu-miR-208b-3p	0	0	-1.60171E-16	1
mmu-miR-208b-5p	0	0	-1.60171E-16	1
mmu-miR-211-3p	0	0	-1.60171E-16	1
mmu-miR-2136	0	0	-1.60171E-16	1
mmu-miR-2139	0	0	-1.60171E-16	1
mmu-miR-214-3p	0	0	-1.60171E-16	1
mmu-miR-214-5p	0	0	-1.60171E-16	1
mmu-miR-215-3p	0	0	-1.60171E-16	1
mmu-miR-216a-3p	0	0	-1.60171E-16	1
mmu-miR-216a-5p	0	0	-1.60171E-16	1
mmu-miR-216b-3p	0	0	-1.60171E-16	1
mmu-miR-216b-5p	0	0	-1.60171E-16	1
mmu-miR-216c-3p	0	0	-1.60171E-16	1
mmu-miR-216c-5p	0	0	-1.60171E-16	1
mmu-miR-217-3p	0	0	-1.60171E-16	1
mmu-miR-217-5p	0	0	-1.60171E-16	1
mmu-miR-218-1-3p	0	0	-1.60171E-16	1
mmu-miR-218-2-3p	0	0	-1.60171E-16	1
mmu-miR-2183	0	0	-1.60171E-16	1
mmu-miR-219a-2-3p	0	0	-1.60171E-16	1
mmu-miR-224-3p	0	0	-1.60171E-16	1
mmu-miR-224-5p	0	0	-1.60171E-16	1
mmu-miR-2861	0	0	-1.60171E-16	1
mmu-miR-290a-3p	0	0	-1.60171E-16	1
mmu-miR-290a-5p	0	0	-1.60171E-16	1
mmu-miR-290b-3p	0	0	-1.60171E-16	1
mmu-miR-290b-5p	0	0	-1.60171E-16	1
mmu-miR-291a-3p	0	0	-1.60171E-16	1
mmu-miR-291a-5p	0	0	-1.60171E-16	1

mmu-miR-291b-3p	0	0	-1.60171E-16	1
mmu-miR-291b-5p	0	0	-1.60171E-16	1
mmu-miR-292a-3p	0	0	-1.60171E-16	1
mmu-miR-292a-5p	0	0	-1.60171E-16	1
mmu-miR-292b-3p	0	0	-1.60171E-16	1
mmu-miR-292b-5p	0	0	-1.60171E-16	1
mmu-miR-293-3p	0	0	-1.60171E-16	1
mmu-miR-293-5p	0	0	-1.60171E-16	1
mmu-miR-294-3p	0	0	-1.60171E-16	1
mmu-miR-294-5p	0	0	-1.60171E-16	1
mmu-miR-295-3p	0	0	-1.60171E-16	1
mmu-miR-295-5p	0	0	-1.60171E-16	1
mmu-miR-297c-5p	1.444284731	0.787089063	-2.22045E-16	1
mmu-miR-299b-3p	0	0	-1.60171E-16	1
mmu-miR-299b-5p	0	0	-1.60171E-16	1
mmu-miR-300-5p	0	0	-1.60171E-16	1
mmu-miR-302a-3p	0	0	-1.60171E-16	1
mmu-miR-302a-5p	0	0	-1.60171E-16	1
mmu-miR-302b-3p	0	0	-1.60171E-16	1
mmu-miR-302b-5p	0	0	-1.60171E-16	1
mmu-miR-302c-3p	0	0	-1.60171E-16	1
mmu-miR-302c-5p	0	0	-1.60171E-16	1
mmu-miR-302d-3p	0	0	-1.60171E-16	1
mmu-miR-302d-5p	0	0	-1.60171E-16	1
mmu-miR-3057-3p	0	0	-1.60171E-16	1
mmu-miR-3059-3p	0	0	-1.60171E-16	1
mmu-miR-3059-5p	0	0	-1.60171E-16	1
mmu-miR-3060-5p	0	0	-1.60171E-16	1
mmu-miR-3062-3p	0	0	-1.60171E-16	1
mmu-miR-3063-3p	0	0	-1.60171E-16	1
mmu-miR-3063-5p	0	0	-1.60171E-16	1
mmu-miR-3065-3p	0	0	-1.60171E-16	1
mmu-miR-3065-5p	0	0	-1.60171E-16	1
mmu-miR-3067-3p	0	0	-1.60171E-16	1
mmu-miR-3067-5p	0	0	-1.60171E-16	1
mmu-miR-3069-5p	0	0	-1.60171E-16	1
mmu-miR-3070-2-3p	0	0	-1.60171E-16	1
mmu-miR-3070-3p	0	0	-1.60171E-16	1
mmu-miR-3070-5p	0	0	-1.60171E-16	1

mmu-miR-3071-3p	0	0	-1.60171E-16	1
mmu-miR-3071-5p	0	0	-1.60171E-16	1
mmu-miR-3072-3p	0	0	-1.60171E-16	1
mmu-miR-3072-5p	0	0	-1.60171E-16	1
mmu-miR-3073a-3p	0	0	-1.60171E-16	1
mmu-miR-3073a-5p	0	0	-1.60171E-16	1
mmu-miR-3073b-3p	0	0	-1.60171E-16	1
mmu-miR-3073b-5p	0	0	-1.60171E-16	1
mmu-miR-3074-2-3p	0	0	-1.60171E-16	1
mmu-miR-3075-3p	0	0	-1.60171E-16	1
mmu-miR-3076-5p	0	0	-1.60171E-16	1
mmu-miR-3077-5p	0	0	-1.60171E-16	1
mmu-miR-3078-3p	0	0	-1.60171E-16	1
mmu-miR-3078-5p	0	0	-1.60171E-16	1
mmu-miR-3080-5p	0	0	-1.60171E-16	1
mmu-miR-3081-3p	0	0	-1.60171E-16	1
mmu-miR-3081-5p	0	0	-1.60171E-16	1
mmu-miR-3082-5p	0	0	-1.60171E-16	1
mmu-miR-3083-3p	0	0	-1.60171E-16	1
mmu-miR-3083-5p	0	0	-1.60171E-16	1
mmu-miR-3085-3p	0	0	-1.60171E-16	1
mmu-miR-3085-5p	0	0	-1.60171E-16	1
mmu-miR-3086-3p	0	0	-1.60171E-16	1
mmu-miR-3086-5p	0.722142365	0.393544532	-1.11022E-16	1
mmu-miR-3088-3p	0	0	-1.60171E-16	1
mmu-miR-3088-5p	0	0	-1.60171E-16	1
mmu-miR-3089-3p	0	0	-1.60171E-16	1
mmu-miR-3089-5p	0	0	-1.60171E-16	1
mmu-miR-3090-3p	0	0	-1.60171E-16	1
mmu-miR-3090-5p	0	0	-1.60171E-16	1
mmu-miR-3092-5p	0	0	-1.60171E-16	1
mmu-miR-3093-3p	0	0	-1.60171E-16	1
mmu-miR-3093-5p	0	0	-1.60171E-16	1
mmu-miR-3095-3p	0	0	-1.60171E-16	1
mmu-miR-3095-5p	0	0	-1.60171E-16	1
mmu-miR-3097-5p	0	0	-1.60171E-16	1
mmu-miR-3099-3p	0	0	-1.60171E-16	1
mmu-miR-3099-5p	0	0	-1.60171E-16	1
mmu-miR-30f	0.361071183	0.196772266	-1.60171E-16	1

mmu-miR-3100-3p	0	0	-1.60171E-16	1
mmu-miR-3100-5p	0	0	-1.60171E-16	1
mmu-miR-3102-5p	0	0	-1.60171E-16	1
mmu-miR-3103-5p	0.361071183	0.196772266	-1.60171E-16	1
mmu-miR-3104-3p	0	0	-1.60171E-16	1
mmu-miR-3104-5p	0	0	-1.60171E-16	1
mmu-miR-3105-3p	0	0	-1.60171E-16	1
mmu-miR-3105-5p	0	0	-1.60171E-16	1
mmu-miR-3106-3p	0	0	-1.60171E-16	1
mmu-miR-3106-5p	0	0	-1.60171E-16	1
mmu-miR-3108-3p	0	0	-1.60171E-16	1
mmu-miR-3110-5p	0.361071183	0.196772266	-1.60171E-16	1
mmu-miR-3113-3p	0	0	-1.60171E-16	1
mmu-miR-3113-5p	0	0	-1.60171E-16	1
mmu-miR-3154	0	0	-1.60171E-16	1
mmu-miR-320-5p	0	0	-1.60171E-16	1
mmu-miR-323-5p	0	0	-1.60171E-16	1
mmu-miR-325-3p	0	0	-1.60171E-16	1
mmu-miR-325-5p	0	0	-1.60171E-16	1
mmu-miR-327	0	0	-1.60171E-16	1
mmu-miR-329-3p	0	0	-1.60171E-16	1
mmu-miR-329-5p	0.361071183	0.196772266	-1.60171E-16	1
mmu-miR-337-3p	0	0	-1.60171E-16	1
mmu-miR-337-5p	0	0	-1.60171E-16	1
mmu-miR-341-5p	0	0	-1.60171E-16	1
mmu-miR-343	0	0	-1.60171E-16	1
mmu-miR-344-3p	0	0	-1.60171E-16	1
mmu-miR-344-5p	0	0	-1.60171E-16	1
mmu-miR-344b-3p	0	0	-1.60171E-16	1
mmu-miR-344b-5p	0	0	-1.60171E-16	1
mmu-miR-344c-3p	0	0	-1.60171E-16	1
mmu-miR-344c-5p	0	0	-1.60171E-16	1
mmu-miR-344d-1-5p	0	0	-1.60171E-16	1
mmu-miR-344d-2-5p	0	0	-1.60171E-16	1
mmu-miR-344d-3-5p	0	0	-1.60171E-16	1
mmu-miR-344d-3p	0	0	-1.60171E-16	1
mmu-miR-344e-3p	0	0	-1.60171E-16	1
mmu-miR-344e-5p	0	0	-1.60171E-16	1
mmu-miR-344f-3p	0	0	-1.60171E-16	1

mmu-miR-344f-5p	0	0	-1.60171E-16	1
mmu-miR-344g-3p	0	0	-1.60171E-16	1
mmu-miR-344g-5p	0	0	-1.60171E-16	1
mmu-miR-344h-3p	0	0	-1.60171E-16	1
mmu-miR-344h-5p	0	0	-1.60171E-16	1
mmu-miR-344i	0	0	-1.60171E-16	1
mmu-miR-346-5p	0	0	-1.60171E-16	1
mmu-miR-3473c	0.361071183	0.196772266	-1.60171E-16	1
mmu-miR-3474	0	0	-1.60171E-16	1
mmu-miR-3475-3p	0	0	-1.60171E-16	1
mmu-miR-3475-5p	0	0	-1.60171E-16	1
mmu-miR-34c-3p	0	0	-1.60171E-16	1
mmu-miR-3544-3p	0	0	-1.60171E-16	1
mmu-miR-3544-5p	0	0	-1.60171E-16	1
mmu-miR-3547-3p	0	0	-1.60171E-16	1
mmu-miR-3547-5p	0	0	-1.60171E-16	1
mmu-miR-3552	0	0	-1.60171E-16	1
mmu-miR-3569-3p	0	0	-1.60171E-16	1
mmu-miR-3569-5p	0.361071183	0.196772266	-1.60171E-16	1
mmu-miR-3618-3p	0	0	-1.60171E-16	1
mmu-miR-3620-3p	0	0	-1.60171E-16	1
mmu-miR-3620-5p	0	0	-1.60171E-16	1
mmu-miR-365-1-5p	0	0	-1.60171E-16	1
mmu-miR-367-3p	0	0	-1.60171E-16	1
mmu-miR-367-5p	0	0	-1.60171E-16	1
mmu-miR-369-5p	0	0	-1.60171E-16	1
mmu-miR-370-3p	0	0	-1.60171E-16	1
mmu-miR-370-5p	0	0	-1.60171E-16	1
mmu-miR-374b-3p	0	0	-1.60171E-16	1
mmu-miR-374c-5p	0	0	-1.60171E-16	1
mmu-miR-375-5p	0	0	-1.60171E-16	1
mmu-miR-376a-3p	0	0	-1.60171E-16	1
mmu-miR-376a-5p	0	0	-1.60171E-16	1
mmu-miR-376b-3p	0	0	-1.60171E-16	1
mmu-miR-376b-5p	0	0	-1.60171E-16	1
mmu-miR-376c-3p	0	0	-1.60171E-16	1
mmu-miR-376c-5p	0	0	-1.60171E-16	1
mmu-miR-377-3p	0	0	-1.60171E-16	1
mmu-miR-377-5p	0	0	-1.60171E-16	1

mmu-miR-380-5p	0	0	-1.60171E-16	1
mmu-miR-381-5p	0	0	-1.60171E-16	1
mmu-miR-382-3p	0	0	-1.60171E-16	1
mmu-miR-382-5p	0	0	-1.60171E-16	1
mmu-miR-383-3p	0	0	-1.60171E-16	1
mmu-miR-383-5p	0	0	-1.60171E-16	1
mmu-miR-384-3p	0	0	-1.60171E-16	1
mmu-miR-384-5p	0	0	-1.60171E-16	1
mmu-miR-3961	0	0	-1.60171E-16	1
mmu-miR-3965	0	0	-1.60171E-16	1
mmu-miR-3967	0	0	-1.60171E-16	1
mmu-miR-3971	0	0	-1.60171E-16	1
mmu-miR-409-5p	0	0	-1.60171E-16	1
mmu-miR-410-5p	0	0	-1.60171E-16	1
mmu-miR-412-3p	0	0	-1.60171E-16	1
mmu-miR-412-5p	0	0	-1.60171E-16	1
mmu-miR-429-5p	0	0	-1.60171E-16	1
mmu-miR-432	0	0	-1.60171E-16	1
mmu-miR-448-3p	0	0	-1.60171E-16	1
mmu-miR-448-5p	0	0	-1.60171E-16	1
mmu-miR-449c-3p	0	0	-1.60171E-16	1
mmu-miR-450a-1-3p	0	0	-1.60171E-16	1
mmu-miR-451b	0	0	-1.60171E-16	1
mmu-miR-452-3p	0	0	-1.60171E-16	1
mmu-miR-452-5p	0	0	-1.60171E-16	1
mmu-miR-453	0	0	-1.60171E-16	1
mmu-miR-463-3p	0	0	-1.60171E-16	1
mmu-miR-463-5p	0	0	-1.60171E-16	1
mmu-miR-465a-5p	0	0	-1.60171E-16	1
mmu-miR-465b-5p	0	0	-1.60171E-16	1
mmu-miR-465d-3p	0	0	-1.60171E-16	1
mmu-miR-465d-5p	0	0	-1.60171E-16	1
mmu-miR-466l-5p	0	0	-1.60171E-16	1
mmu-miR-467f	0	0	-1.60171E-16	1
mmu-miR-467g	0	0	-1.60171E-16	1
mmu-miR-468-3p	0	0	-1.60171E-16	1
mmu-miR-468-5p	0	0	-1.60171E-16	1
mmu-miR-470-3p	0	0	-1.60171E-16	1
mmu-miR-471-3p	0	0	-1.60171E-16	1

mmu-miR-471-5p	0	0	-1.60171E-16	1
mmu-miR-483-3p	0	0	-1.60171E-16	1
mmu-miR-483-5p	0	0	-1.60171E-16	1
mmu-miR-487b-5p	0	0	-1.60171E-16	1
mmu-miR-488-3p	0	0	-1.60171E-16	1
mmu-miR-488-5p	0	0	-1.60171E-16	1
mmu-miR-489-3p	0	0	-1.60171E-16	1
mmu-miR-489-5p	0	0	-1.60171E-16	1
mmu-miR-490-3p	0	0	-1.60171E-16	1
mmu-miR-490-5p	0	0	-1.60171E-16	1
mmu-miR-493-5p	0	0	-1.60171E-16	1
mmu-miR-494-3p	0	0	-1.60171E-16	1
mmu-miR-494-5p	0	0	-1.60171E-16	1
mmu-miR-495-3p	0.361071183	0.196772266	-1.60171E-16	1
mmu-miR-495-5p	0	0	-1.60171E-16	1
mmu-miR-496a-3p	0	0	-1.60171E-16	1
mmu-miR-496a-5p	0	0	-1.60171E-16	1
mmu-miR-496b	0	0	-1.60171E-16	1
mmu-miR-497a-3p	0	0	-1.60171E-16	1
mmu-miR-497b	0	0	-1.60171E-16	1
mmu-miR-499-3p	0	0	-1.60171E-16	1
mmu-miR-499-5p	0	0	-1.60171E-16	1
mmu-miR-5046	0	0	-1.60171E-16	1
mmu-miR-509-3p	0	0	-1.60171E-16	1
mmu-miR-509-5p	0	0	-1.60171E-16	1
mmu-miR-5098	0	0	-1.60171E-16	1
mmu-miR-5101	0	0	-1.60171E-16	1
mmu-miR-5108	0	0	-1.60171E-16	1
mmu-miR-511-3p	0	0	-1.60171E-16	1
mmu-miR-511-5p	0	0	-1.60171E-16	1
mmu-miR-5110	0	0	-1.60171E-16	1
mmu-miR-5112	0	0	-1.60171E-16	1
mmu-miR-5118	0	0	-1.60171E-16	1
mmu-miR-5124a	0	0	-1.60171E-16	1
mmu-miR-5124b	0	0	-1.60171E-16	1
mmu-miR-5125	0	0	-1.60171E-16	1
mmu-miR-5129-3p	0	0	-1.60171E-16	1
mmu-miR-5129-5p	0	0	-1.60171E-16	1
mmu-miR-5130	0	0	-1.60171E-16	1

mmu-miR-5131	0	0	-1.60171E-16	1
mmu-miR-5134-3p	0.361071183	0.196772266	-1.60171E-16	1
mmu-miR-5134-5p	0.361071183	0.196772266	-1.60171E-16	1
mmu-miR-5135	0	0	-1.60171E-16	1
mmu-miR-5136	0	0	-1.60171E-16	1
mmu-miR-539-3p	0	0	-1.60171E-16	1
mmu-miR-539-5p	0	0	-1.60171E-16	1
mmu-miR-540-5p	0	0	-1.60171E-16	1
mmu-miR-541-3p	0	0	-1.60171E-16	1
mmu-miR-543-5p	0	0	-1.60171E-16	1
mmu-miR-544-3p	0	0	-1.60171E-16	1
mmu-miR-544-5p	0	0	-1.60171E-16	1
mmu-miR-546	0	0	-1.60171E-16	1
mmu-miR-547-3p	0	0	-1.60171E-16	1
mmu-miR-547-5p	0	0	-1.60171E-16	1
mmu-miR-551b-3p	0	0	-1.60171E-16	1
mmu-miR-551b-5p	0	0	-1.60171E-16	1
mmu-miR-5616-5p	0	0	-1.60171E-16	1
mmu-miR-5617-3p	0	0	-1.60171E-16	1
mmu-miR-5617-5p	0	0	-1.60171E-16	1
mmu-miR-5618-3p	0	0	-1.60171E-16	1
mmu-miR-5618-5p	0	0	-1.60171E-16	1
mmu-miR-5619-3p	1.444284731	0.787089063	-2.22045E-16	1
mmu-miR-5620-5p	0	0	-1.60171E-16	1
mmu-miR-5621-3p	0	0	-1.60171E-16	1
mmu-miR-5621-5p	0	0	-1.60171E-16	1
mmu-miR-5623-3p	0	0	-1.60171E-16	1
mmu-miR-5623-5p	0	0	-1.60171E-16	1
mmu-miR-5625-3p	0	0	-1.60171E-16	1
mmu-miR-5625-5p	0	0	-1.60171E-16	1
mmu-miR-5627-3p	0.722142365	0.393544532	-1.11022E-16	1
mmu-miR-5627-5p	0	0	-1.60171E-16	1
mmu-miR-568	0	0	-1.60171E-16	1
mmu-miR-5709-3p	0	0	-1.60171E-16	1
mmu-miR-5709-5p	0	0	-1.60171E-16	1
mmu-miR-5710	0	0	-1.60171E-16	1
mmu-miR-582-5p	0	0	-1.60171E-16	1
mmu-miR-590-3p	0	0	-1.60171E-16	1
mmu-miR-590-5p	0	0	-1.60171E-16	1

mmu-miR-592-3p	0	0	-1.60171E-16	1
mmu-miR-598-3p	0	0	-1.60171E-16	1
mmu-miR-598-5p	0	0	-1.60171E-16	1
mmu-miR-599	0	0	-1.60171E-16	1
mmu-miR-615-3p	0	0	-1.60171E-16	1
mmu-miR-615-5p	0	0	-1.60171E-16	1
mmu-miR-6237	0	0	-1.60171E-16	1
mmu-miR-6239	0	0	-1.60171E-16	1
mmu-miR-6241	0	0	-1.60171E-16	1
mmu-miR-6244	0	0	-1.60171E-16	1
mmu-miR-6335	0	0	-1.60171E-16	1
mmu-miR-6336	0	0	-1.60171E-16	1
mmu-miR-6337	0	0	-1.60171E-16	1
mmu-miR-6338	0	0	-1.60171E-16	1
mmu-miR-6339	0	0	-1.60171E-16	1
mmu-miR-6340	0	0	-1.60171E-16	1
mmu-miR-6341	0	0	-1.60171E-16	1
mmu-miR-6342	0	0	-1.60171E-16	1
mmu-miR-6343	0	0	-1.60171E-16	1
mmu-miR-6344	0	0	-1.60171E-16	1
mmu-miR-6345	0	0	-1.60171E-16	1
mmu-miR-6346	0	0	-1.60171E-16	1
mmu-miR-6347	0	0	-1.60171E-16	1
mmu-miR-6348	0	0	-1.60171E-16	1
mmu-miR-6349	0	0	-1.60171E-16	1
mmu-miR-6350	0	0	-1.60171E-16	1
mmu-miR-6351	0	0	-1.60171E-16	1
mmu-miR-6352	0	0	-1.60171E-16	1
mmu-miR-6354	0	0	-1.60171E-16	1
mmu-miR-6355	0	0	-1.60171E-16	1
mmu-miR-6356	0	0	-1.60171E-16	1
mmu-miR-6357	0	0	-1.60171E-16	1
mmu-miR-6358	0	0	-1.60171E-16	1
mmu-miR-6359	0	0	-1.60171E-16	1
mmu-miR-6360	0	0	-1.60171E-16	1
mmu-miR-6361	0	0	-1.60171E-16	1
mmu-miR-6362	0	0	-1.60171E-16	1
mmu-miR-6363	0	0	-1.60171E-16	1
mmu-miR-6364	0	0	-1.60171E-16	1

mmu-miR-6365	0	0	-1.60171E-16	1
mmu-miR-6366	0	0	-1.60171E-16	1
mmu-miR-6367	0	0	-1.60171E-16	1
mmu-miR-6368	0	0	-1.60171E-16	1
mmu-miR-6369	0	0	-1.60171E-16	1
mmu-miR-6370	0	0	-1.60171E-16	1
mmu-miR-6371	0	0	-1.60171E-16	1
mmu-miR-6372	0	0	-1.60171E-16	1
mmu-miR-6373	0	0	-1.60171E-16	1
mmu-miR-6374	0	0	-1.60171E-16	1
mmu-miR-6375	0	0	-1.60171E-16	1
mmu-miR-6376	0	0	-1.60171E-16	1
mmu-miR-6377	0	0	-1.60171E-16	1
mmu-miR-6378	0	0	-1.60171E-16	1
mmu-miR-6379	0	0	-1.60171E-16	1
mmu-miR-6381	0	0	-1.60171E-16	1
mmu-miR-6382	0	0	-1.60171E-16	1
mmu-miR-6383	0	0	-1.60171E-16	1
mmu-miR-6384	0	0	-1.60171E-16	1
mmu-miR-6385	0	0	-1.60171E-16	1
mmu-miR-6386	0	0	-1.60171E-16	1
mmu-miR-6387	0	0	-1.60171E-16	1
mmu-miR-6388	0	0	-1.60171E-16	1
mmu-miR-6389	0	0	-1.60171E-16	1
mmu-miR-6390	0	0	-1.60171E-16	1
mmu-miR-6391	0	0	-1.60171E-16	1
mmu-miR-6392-3p	0	0	-1.60171E-16	1
mmu-miR-6392-5p	0	0	-1.60171E-16	1
mmu-miR-6393	0	0	-1.60171E-16	1
mmu-miR-6394	0	0	-1.60171E-16	1
mmu-miR-6396	0	0	-1.60171E-16	1
mmu-miR-6397	0	0	-1.60171E-16	1
mmu-miR-6398	0	0	-1.60171E-16	1
mmu-miR-6400	0	0	-1.60171E-16	1
mmu-miR-6401	0	0	-1.60171E-16	1
mmu-miR-6402	0	0	-1.60171E-16	1
mmu-miR-6403	0	0	-1.60171E-16	1
mmu-miR-6404	0	0	-1.60171E-16	1
mmu-miR-6405	0	0	-1.60171E-16	1

mmu-miR-6406	0	0	-1.60171E-16	1
mmu-miR-6407	0	0	-1.60171E-16	1
mmu-miR-6408	0	0	-1.60171E-16	1
mmu-miR-6409	0	0	-1.60171E-16	1
mmu-miR-6410	0	0	-1.60171E-16	1
mmu-miR-6411	0	0	-1.60171E-16	1
mmu-miR-6413	0	0	-1.60171E-16	1
mmu-miR-6414	0	0	-1.60171E-16	1
mmu-miR-6415	0	0	-1.60171E-16	1
mmu-miR-6416-3p	0	0	-1.60171E-16	1
mmu-miR-6416-5p	0	0	-1.60171E-16	1
mmu-miR-6417	0	0	-1.60171E-16	1
mmu-miR-6418-5p	0	0	-1.60171E-16	1
mmu-miR-6419	0	0	-1.60171E-16	1
mmu-miR-6420	0	0	-1.60171E-16	1
mmu-miR-6481	0.361071183	0.196772266	-1.60171E-16	1
mmu-miR-653-3p	0	0	-1.60171E-16	1
mmu-miR-654-3p	0	0	-1.60171E-16	1
mmu-miR-654-5p	0	0	-1.60171E-16	1
mmu-miR-6540-3p	0	0	-1.60171E-16	1
mmu-miR-6540-5p	0	0	-1.60171E-16	1
mmu-miR-665-5p	0	0	-1.60171E-16	1
mmu-miR-666-3p	0	0	-1.60171E-16	1
mmu-miR-668-5p	0	0	-1.60171E-16	1
mmu-miR-669c-3p	0	0	-1.60171E-16	1
mmu-miR-669g	0.361071183	0.196772266	-1.60171E-16	1
mmu-miR-669i	0	0	-1.60171E-16	1
mmu-miR-669j	0	0	-1.60171E-16	1
mmu-miR-669k-3p	0	0	-1.60171E-16	1
mmu-miR-669n	0	0	-1.60171E-16	1
mmu-miR-670-3p	0	0	-1.60171E-16	1
mmu-miR-670-5p	0	0	-1.60171E-16	1
mmu-miR-6715-3p	0	0	-1.60171E-16	1
mmu-miR-6715-5p	0	0	-1.60171E-16	1
mmu-miR-672-3p	0	0	-1.60171E-16	1
mmu-miR-675-3p	0	0	-1.60171E-16	1
mmu-miR-675-5p	0	0	-1.60171E-16	1
mmu-miR-6769b-3p	0	0	-1.60171E-16	1
mmu-miR-6769b-5p	0	0	-1.60171E-16	1

mmu-miR-677-3p	0	0	-1.60171E-16	1
mmu-miR-678	0	0	-1.60171E-16	1
mmu-miR-679-3p	0	0	-1.60171E-16	1
mmu-miR-680	0	0	-1.60171E-16	1
mmu-miR-681	0	0	-1.60171E-16	1
mmu-miR-683	0	0	-1.60171E-16	1
mmu-miR-684	0	0	-1.60171E-16	1
mmu-miR-687	0	0	-1.60171E-16	1
mmu-miR-688	0	0	-1.60171E-16	1
mmu-miR-6896-3p	0	0	-1.60171E-16	1
mmu-miR-6896-5p	0	0	-1.60171E-16	1
mmu-miR-6897-3p	0	0	-1.60171E-16	1
mmu-miR-6897-5p	0	0	-1.60171E-16	1
mmu-miR-6898-3p	0	0	-1.60171E-16	1
mmu-miR-6899-5p	0	0	-1.60171E-16	1
mmu-miR-6901-3p	0	0	-1.60171E-16	1
mmu-miR-6901-5p	0	0	-1.60171E-16	1
mmu-miR-6902-3p	0	0	-1.60171E-16	1
mmu-miR-6903-3p	0	0	-1.60171E-16	1
mmu-miR-6903-5p	0	0	-1.60171E-16	1
mmu-miR-6904-5p	0	0	-1.60171E-16	1
mmu-miR-6905-3p	0	0	-1.60171E-16	1
mmu-miR-6905-5p	0	0	-1.60171E-16	1
mmu-miR-6906-3p	0	0	-1.60171E-16	1
mmu-miR-6906-5p	0	0	-1.60171E-16	1
mmu-miR-6907-3p	0	0	-1.60171E-16	1
mmu-miR-6907-5p	0	0	-1.60171E-16	1
mmu-miR-6908-3p	0	0	-1.60171E-16	1
mmu-miR-6909-3p	0	0	-1.60171E-16	1
mmu-miR-6909-5p	0	0	-1.60171E-16	1
mmu-miR-691	0	0	-1.60171E-16	1
mmu-miR-6911-5p	0	0	-1.60171E-16	1
mmu-miR-6912-3p	0	0	-1.60171E-16	1
mmu-miR-6912-5p	0	0	-1.60171E-16	1
mmu-miR-6913-5p	0	0	-1.60171E-16	1
mmu-miR-6914-5p	0	0	-1.60171E-16	1
mmu-miR-6915-5p	0	0	-1.60171E-16	1
mmu-miR-6916-3p	0	0	-1.60171E-16	1
mmu-miR-6917-3p	0	0	-1.60171E-16	1

mmu-miR-6917-5p	0	0	-1.60171E-16	1
mmu-miR-6918-5p	0	0	-1.60171E-16	1
mmu-miR-6919-3p	0	0	-1.60171E-16	1
mmu-miR-6919-5p	0	0	-1.60171E-16	1
mmu-miR-6920-3p	0	0	-1.60171E-16	1
mmu-miR-6920-5p	0	0	-1.60171E-16	1
mmu-miR-6921-3p	0	0	-1.60171E-16	1
mmu-miR-6921-5p	0	0	-1.60171E-16	1
mmu-miR-6923-3p	0	0	-1.60171E-16	1
mmu-miR-6923-5p	0	0	-1.60171E-16	1
mmu-miR-6925-3p	0	0	-1.60171E-16	1
mmu-miR-6926-5p	0	0	-1.60171E-16	1
mmu-miR-6927-5p	0	0	-1.60171E-16	1
mmu-miR-6928-3p	0	0	-1.60171E-16	1
mmu-miR-6928-5p	0	0	-1.60171E-16	1
mmu-miR-6929-3p	0	0	-1.60171E-16	1
mmu-miR-6929-5p	0	0	-1.60171E-16	1
mmu-miR-693-5p	0	0	-1.60171E-16	1
mmu-miR-6930-3p	0	0	-1.60171E-16	1
mmu-miR-6930-5p	0	0	-1.60171E-16	1
mmu-miR-6931-3p	0	0	-1.60171E-16	1
mmu-miR-6931-5p	0	0	-1.60171E-16	1
mmu-miR-6932-3p	0	0	-1.60171E-16	1
mmu-miR-6933-3p	0	0	-1.60171E-16	1
mmu-miR-6934-3p	0	0	-1.60171E-16	1
mmu-miR-6934-5p	0	0	-1.60171E-16	1
mmu-miR-6935-3p	0	0	-1.60171E-16	1
mmu-miR-6936-3p	0	0	-1.60171E-16	1
mmu-miR-6936-5p	0	0	-1.60171E-16	1
mmu-miR-6937-5p	0	0	-1.60171E-16	1
mmu-miR-6938-5p	0	0	-1.60171E-16	1
mmu-miR-6939-3p	0	0	-1.60171E-16	1
mmu-miR-6939-5p	0	0	-1.60171E-16	1
mmu-miR-694	0	0	-1.60171E-16	1
mmu-miR-6940-3p	0	0	-1.60171E-16	1
mmu-miR-6940-5p	0	0	-1.60171E-16	1
mmu-miR-6941-3p	0	0	-1.60171E-16	1
mmu-miR-6941-5p	0	0	-1.60171E-16	1
mmu-miR-6942-3p	0	0	-1.60171E-16	1

mmu-miR-6944-5p	0	0	-1.60171E-16	1
mmu-miR-6945-3p	0	0	-1.60171E-16	1
mmu-miR-6945-5p	0	0	-1.60171E-16	1
mmu-miR-6947-5p	0	0	-1.60171E-16	1
mmu-miR-6949-3p	0	0	-1.60171E-16	1
mmu-miR-6949-5p	0	0	-1.60171E-16	1
mmu-miR-695	0	0	-1.60171E-16	1
mmu-miR-6950-3p	0	0	-1.60171E-16	1
mmu-miR-6950-5p	0	0	-1.60171E-16	1
mmu-miR-6951-3p	0	0	-1.60171E-16	1
mmu-miR-6951-5p	0	0	-1.60171E-16	1
mmu-miR-6953-3p	0	0	-1.60171E-16	1
mmu-miR-6953-5p	0	0	-1.60171E-16	1
mmu-miR-6954-3p	0	0	-1.60171E-16	1
mmu-miR-6954-5p	0	0	-1.60171E-16	1
mmu-miR-6956-5p	0	0	-1.60171E-16	1
mmu-miR-6957-3p	0	0	-1.60171E-16	1
mmu-miR-6958-5p	0	0	-1.60171E-16	1
mmu-miR-6959-3p	0	0	-1.60171E-16	1
mmu-miR-696	0	0	-1.60171E-16	1
mmu-miR-6960-3p	0	0	-1.60171E-16	1
mmu-miR-6961-3p	0	0	-1.60171E-16	1
mmu-miR-6963-3p	0	0	-1.60171E-16	1
mmu-miR-6963-5p	0	0	-1.60171E-16	1
mmu-miR-6964-5p	0	0	-1.60171E-16	1
mmu-miR-6965-5p	0	0	-1.60171E-16	1
mmu-miR-6966-5p	0	0	-1.60171E-16	1
mmu-miR-6968-3p	0	0	-1.60171E-16	1
mmu-miR-6968-5p	0	0	-1.60171E-16	1
mmu-miR-6969-3p	0	0	-1.60171E-16	1
mmu-miR-6969-5p	0	0	-1.60171E-16	1
mmu-miR-697	0	0	-1.60171E-16	1
mmu-miR-6970-5p	1.444284731	0.787089063	-2.22045E-16	1
mmu-miR-6971-5p	0	0	-1.60171E-16	1
mmu-miR-6972-3p	0	0	-1.60171E-16	1
mmu-miR-6972-5p	0	0	-1.60171E-16	1
mmu-miR-6973a-5p	0	0	-1.60171E-16	1
mmu-miR-6973b-3p	0	0	-1.60171E-16	1
mmu-miR-6973b-5p	0	0	-1.60171E-16	1

mmu-miR-6975-5p	0	0	-1.60171E-16	1
mmu-miR-6976-3p	0	0	-1.60171E-16	1
mmu-miR-6976-5p	0	0	-1.60171E-16	1
mmu-miR-6977-3p	0	0	-1.60171E-16	1
mmu-miR-6978-3p	0	0	-1.60171E-16	1
mmu-miR-6978-5p	0	0	-1.60171E-16	1
mmu-miR-6979-5p	0	0	-1.60171E-16	1
mmu-miR-698-3p	0	0	-1.60171E-16	1
mmu-miR-698-5p	0	0	-1.60171E-16	1
mmu-miR-6980-3p	0	0	-1.60171E-16	1
mmu-miR-6980-5p	0	0	-1.60171E-16	1
mmu-miR-6981-3p	0	0	-1.60171E-16	1
mmu-miR-6981-5p	0	0	-1.60171E-16	1
mmu-miR-6982-3p	0	0	-1.60171E-16	1
mmu-miR-6982-5p	0	0	-1.60171E-16	1
mmu-miR-6983-3p	0	0	-1.60171E-16	1
mmu-miR-6983-5p	0	0	-1.60171E-16	1
mmu-miR-6984-5p	0	0	-1.60171E-16	1
mmu-miR-6985-5p	0	0	-1.60171E-16	1
mmu-miR-6986-3p	0	0	-1.60171E-16	1
mmu-miR-6987-3p	0	0	-1.60171E-16	1
mmu-miR-6988-5p	0	0	-1.60171E-16	1
mmu-miR-6989-5p	0	0	-1.60171E-16	1
mmu-miR-6991-3p	0	0	-1.60171E-16	1
mmu-miR-6991-5p	0	0	-1.60171E-16	1
mmu-miR-6993-3p	0	0	-1.60171E-16	1
mmu-miR-6993-5p	0	0	-1.60171E-16	1
mmu-miR-6995-3p	0	0	-1.60171E-16	1
mmu-miR-6995-5p	0	0	-1.60171E-16	1
mmu-miR-6996-3p	0	0	-1.60171E-16	1
mmu-miR-6998-5p	0	0	-1.60171E-16	1
mmu-miR-7000-3p	0	0	-1.60171E-16	1
mmu-miR-7000-5p	0	0	-1.60171E-16	1
mmu-miR-7001-3p	0	0	-1.60171E-16	1
mmu-miR-7001-5p	0	0	-1.60171E-16	1
mmu-miR-7002-3p	0	0	-1.60171E-16	1
mmu-miR-7002-5p	0	0	-1.60171E-16	1
mmu-miR-7003-3p	0	0	-1.60171E-16	1
mmu-miR-7003-5p	0	0	-1.60171E-16	1

mmu-miR-7004-3p	0	0	-1.60171E-16	1
mmu-miR-7005-3p	0	0	-1.60171E-16	1
mmu-miR-7005-5p	0	0	-1.60171E-16	1
mmu-miR-7006-3p	0	0	-1.60171E-16	1
mmu-miR-7006-5p	0	0	-1.60171E-16	1
mmu-miR-7007-3p	0	0	-1.60171E-16	1
mmu-miR-7007-5p	0	0	-1.60171E-16	1
mmu-miR-7008-3p	0	0	-1.60171E-16	1
mmu-miR-7008-5p	0	0	-1.60171E-16	1
mmu-miR-7009-3p	0	0	-1.60171E-16	1
mmu-miR-7009-5p	0.361071183	0.196772266	-1.60171E-16	1
mmu-miR-7010-3p	0	0	-1.60171E-16	1
mmu-miR-7011-3p	0	0	-1.60171E-16	1
mmu-miR-7011-5p	0	0	-1.60171E-16	1
mmu-miR-7013-3p	0.361071183	0.196772266	-1.60171E-16	1
mmu-miR-7013-5p	0.361071183	0.196772266	-1.60171E-16	1
mmu-miR-7014-3p	0	0	-1.60171E-16	1
mmu-miR-7015-5p	0	0	-1.60171E-16	1
mmu-miR-7016-3p	0	0	-1.60171E-16	1
mmu-miR-7016-5p	0	0	-1.60171E-16	1
mmu-miR-7017-3p	0	0	-1.60171E-16	1
mmu-miR-7018-3p	0	0	-1.60171E-16	1
mmu-miR-7018-5p	0	0	-1.60171E-16	1
mmu-miR-7019-5p	0	0	-1.60171E-16	1
mmu-miR-7020-3p	0	0	-1.60171E-16	1
mmu-miR-7020-5p	0	0	-1.60171E-16	1
mmu-miR-7021-3p	0	0	-1.60171E-16	1
mmu-miR-7022-3p	0.361071183	0.196772266	-1.60171E-16	1
mmu-miR-7022-5p	0	0	-1.60171E-16	1
mmu-miR-7023-5p	0	0	-1.60171E-16	1
mmu-miR-7024-3p	0	0	-1.60171E-16	1
mmu-miR-7024-5p	0	0	-1.60171E-16	1
mmu-miR-7025-3p	0	0	-1.60171E-16	1
mmu-miR-7025-5p	0	0	-1.60171E-16	1
mmu-miR-7026-5p	0	0	-1.60171E-16	1
mmu-miR-7027-3p	0	0	-1.60171E-16	1
mmu-miR-7027-5p	0	0	-1.60171E-16	1
mmu-miR-7028-3p	0	0	-1.60171E-16	1
mmu-miR-7029-3p	0.361071183	0.196772266	-1.60171E-16	1

mmu-miR-703	0	0	-1.60171E-16	1
mmu-miR-7030-5p	0	0	-1.60171E-16	1
mmu-miR-7032-5p	0	0	-1.60171E-16	1
mmu-miR-7033-3p	0	0	-1.60171E-16	1
mmu-miR-7034-3p	0	0	-1.60171E-16	1
mmu-miR-7034-5p	0	0	-1.60171E-16	1
mmu-miR-7035-5p	0	0	-1.60171E-16	1
mmu-miR-7037-3p	0	0	-1.60171E-16	1
mmu-miR-7040-3p	0	0	-1.60171E-16	1
mmu-miR-7040-5p	0	0	-1.60171E-16	1
mmu-miR-7041-3p	0	0	-1.60171E-16	1
mmu-miR-7041-5p	0	0	-1.60171E-16	1
mmu-miR-7043-3p	0	0	-1.60171E-16	1
mmu-miR-7043-5p	0	0	-1.60171E-16	1
mmu-miR-7044-3p	0	0	-1.60171E-16	1
mmu-miR-7044-5p	0	0	-1.60171E-16	1
mmu-miR-7046-5p	0	0	-1.60171E-16	1
mmu-miR-7047-3p	0	0	-1.60171E-16	1
mmu-miR-7047-5p	0	0	-1.60171E-16	1
mmu-miR-7049-3p	0	0	-1.60171E-16	1
mmu-miR-7049-5p	0	0	-1.60171E-16	1
mmu-miR-705	0	0	-1.60171E-16	1
mmu-miR-7050-3p	0	0	-1.60171E-16	1
mmu-miR-7050-5p	0	0	-1.60171E-16	1
mmu-miR-7051-3p	0	0	-1.60171E-16	1
mmu-miR-7052-3p	0	0	-1.60171E-16	1
mmu-miR-7052-5p	0	0	-1.60171E-16	1
mmu-miR-7053-5p	0	0	-1.60171E-16	1
mmu-miR-7055-3p	0	0	-1.60171E-16	1
mmu-miR-7055-5p	0	0	-1.60171E-16	1
mmu-miR-7056-3p	0	0	-1.60171E-16	1
mmu-miR-7056-5p	0	0	-1.60171E-16	1
mmu-miR-7057-3p	0	0	-1.60171E-16	1
mmu-miR-7057-5p	0	0	-1.60171E-16	1
mmu-miR-7059-3p	0	0	-1.60171E-16	1
mmu-miR-706	0	0	-1.60171E-16	1
mmu-miR-7060-3p	0	0	-1.60171E-16	1
mmu-miR-7061-3p	0	0	-1.60171E-16	1
mmu-miR-7062-3p	0	0	-1.60171E-16	1

mmu-miR-7062-5p	0	0	-1.60171E-16	1
mmu-miR-7063-3p	0	0	-1.60171E-16	1
mmu-miR-7064-3p	0	0	-1.60171E-16	1
mmu-miR-7064-5p	0	0	-1.60171E-16	1
mmu-miR-7065-3p	0	0	-1.60171E-16	1
mmu-miR-7065-5p	0	0	-1.60171E-16	1
mmu-miR-7066-3p	0	0	-1.60171E-16	1
mmu-miR-7066-5p	0	0	-1.60171E-16	1
mmu-miR-7067-5p	0	0	-1.60171E-16	1
mmu-miR-7069-3p	0.361071183	0.196772266	-1.60171E-16	1
mmu-miR-707	0	0	-1.60171E-16	1
mmu-miR-7070-3p	0	0	-1.60171E-16	1
mmu-miR-7070-5p	0	0	-1.60171E-16	1
mmu-miR-7071-5p	0	0	-1.60171E-16	1
mmu-miR-7073-3p	0	0	-1.60171E-16	1
mmu-miR-7074-3p	0	0	-1.60171E-16	1
mmu-miR-7074-5p	0	0	-1.60171E-16	1
mmu-miR-7075-5p	0	0	-1.60171E-16	1
mmu-miR-7076-3p	0	0	-1.60171E-16	1
mmu-miR-7076-5p	0	0	-1.60171E-16	1
mmu-miR-7077-3p	0	0	-1.60171E-16	1
mmu-miR-7077-5p	0	0	-1.60171E-16	1
mmu-miR-7078-3p	0	0	-1.60171E-16	1
mmu-miR-7078-5p	0	0	-1.60171E-16	1
mmu-miR-7079-3p	0	0	-1.60171E-16	1
mmu-miR-708-3p	0	0	-1.60171E-16	1
mmu-miR-708-5p	0	0	-1.60171E-16	1
mmu-miR-7080-3p	0	0	-1.60171E-16	1
mmu-miR-7080-5p	0	0	-1.60171E-16	1
mmu-miR-7081-5p	0	0	-1.60171E-16	1
mmu-miR-7082-5p	0	0	-1.60171E-16	1
mmu-miR-7083-3p	0	0	-1.60171E-16	1
mmu-miR-7083-5p	0	0	-1.60171E-16	1
mmu-miR-7084-5p	0	0	-1.60171E-16	1
mmu-miR-7085-5p	0	0	-1.60171E-16	1
mmu-miR-7086-3p	0	0	-1.60171E-16	1
mmu-miR-7086-5p	0	0	-1.60171E-16	1
mmu-miR-7087-3p	0	0	-1.60171E-16	1
mmu-miR-7087-5p	0	0	-1.60171E-16	1

mmu-miR-7088-3p	0	0	-1.60171E-16	1
mmu-miR-7088-5p	0	0	-1.60171E-16	1
mmu-miR-7089-3p	0	0	-1.60171E-16	1
mmu-miR-7090-3p	0	0	-1.60171E-16	1
mmu-miR-7090-5p	0	0	-1.60171E-16	1
mmu-miR-7091-3p	0	0	-1.60171E-16	1
mmu-miR-7092-3p	0	0	-1.60171E-16	1
mmu-miR-7093-3p	0	0	-1.60171E-16	1
mmu-miR-7094-1-5p	0	0	-1.60171E-16	1
mmu-miR-7094-3p	0	0	-1.60171E-16	1
mmu-miR-7094b-2-5p	0	0	-1.60171E-16	1
mmu-miR-710	0	0	-1.60171E-16	1
mmu-miR-711	0	0	-1.60171E-16	1
mmu-miR-7115-3p	0	0	-1.60171E-16	1
mmu-miR-7115-5p	0	0	-1.60171E-16	1
mmu-miR-7116-5p	0	0	-1.60171E-16	1
mmu-miR-7117-3p	0	0	-1.60171E-16	1
mmu-miR-7118-3p	0	0	-1.60171E-16	1
mmu-miR-7118-5p	0	0	-1.60171E-16	1
mmu-miR-7119-3p	0	0	-1.60171E-16	1
mmu-miR-7119-5p	0	0	-1.60171E-16	1
mmu-miR-712-3p	0	0	-1.60171E-16	1
mmu-miR-713	0	0	-1.60171E-16	1
mmu-miR-717	0	0	-1.60171E-16	1
mmu-miR-718	0	0	-1.60171E-16	1
mmu-miR-719	0	0	-1.60171E-16	1
mmu-miR-721	0	0	-1.60171E-16	1
mmu-miR-7210-3p	0	0	-1.60171E-16	1
mmu-miR-7210-5p	0	0	-1.60171E-16	1
mmu-miR-7211-3p	0	0	-1.60171E-16	1
mmu-miR-7211-5p	0	0	-1.60171E-16	1
mmu-miR-7212-5p	0.361071183	0.196772266	-1.60171E-16	1
mmu-miR-7213-3p	0	0	-1.60171E-16	1
mmu-miR-7214-3p	0	0	-1.60171E-16	1
mmu-miR-7214-5p	0	0	-1.60171E-16	1
mmu-miR-7215-3p	0	0	-1.60171E-16	1
mmu-miR-7215-5p	0	0	-1.60171E-16	1
mmu-miR-7216-3p	0	0	-1.60171E-16	1
mmu-miR-7216-5p	0	0	-1.60171E-16	1

mmu-miR-7217-3p	0	0	-1.60171E-16	1
mmu-miR-7217-5p	0	0	-1.60171E-16	1
mmu-miR-7218-3p	0	0	-1.60171E-16	1
mmu-miR-7218-5p	0	0	-1.60171E-16	1
mmu-miR-7219-5p	0	0	-1.60171E-16	1
mmu-miR-7220-3p	0	0	-1.60171E-16	1
mmu-miR-7220-5p	0	0	-1.60171E-16	1
mmu-miR-7221-3p	0	0	-1.60171E-16	1
mmu-miR-7221-5p	0	0	-1.60171E-16	1
mmu-miR-7222-3p	0	0	-1.60171E-16	1
mmu-miR-7222-5p	0	0	-1.60171E-16	1
mmu-miR-7223-3p	0	0	-1.60171E-16	1
mmu-miR-7223-5p	0	0	-1.60171E-16	1
mmu-miR-7224-3p	0	0	-1.60171E-16	1
mmu-miR-7224-5p	0	0	-1.60171E-16	1
mmu-miR-7225-3p	0	0	-1.60171E-16	1
mmu-miR-7225-5p	0	0	-1.60171E-16	1
mmu-miR-7226-3p	0	0	-1.60171E-16	1
mmu-miR-7226-5p	0	0	-1.60171E-16	1
mmu-miR-7227-5p	0	0	-1.60171E-16	1
mmu-miR-7228-3p	0	0	-1.60171E-16	1
mmu-miR-7228-5p	0	0	-1.60171E-16	1
mmu-miR-7229-3p	0	0	-1.60171E-16	1
mmu-miR-7229-5p	0	0	-1.60171E-16	1
mmu-miR-7230-3p	0	0	-1.60171E-16	1
mmu-miR-7230-5p	0	0	-1.60171E-16	1
mmu-miR-7231-3p	0	0	-1.60171E-16	1
mmu-miR-7231-5p	0	0	-1.60171E-16	1
mmu-miR-7232-3p	0	0	-1.60171E-16	1
mmu-miR-7232-5p	0	0	-1.60171E-16	1
mmu-miR-7233-3p	0	0	-1.60171E-16	1
mmu-miR-7233-5p	0	0	-1.60171E-16	1
mmu-miR-7234-3p	0	0	-1.60171E-16	1
mmu-miR-7234-5p	0	0	-1.60171E-16	1
mmu-miR-7235-5p	0	0	-1.60171E-16	1
mmu-miR-7236-3p	0	0	-1.60171E-16	1
mmu-miR-7236-5p	0	0	-1.60171E-16	1
mmu-miR-7237-5p	0	0	-1.60171E-16	1
mmu-miR-7238-3p	0	0	-1.60171E-16	1

mmu-miR-7238-5p	0	0	-1.60171E-16	1
mmu-miR-7239-3p	0	0	-1.60171E-16	1
mmu-miR-7239-5p	0	0	-1.60171E-16	1
mmu-miR-7240-3p	0	0	-1.60171E-16	1
mmu-miR-7241-3p	0	0	-1.60171E-16	1
mmu-miR-7241-5p	0	0	-1.60171E-16	1
mmu-miR-7242-3p	0	0	-1.60171E-16	1
mmu-miR-7242-5p	0	0	-1.60171E-16	1
mmu-miR-7243-3p	0	0	-1.60171E-16	1
mmu-miR-7243-5p	0	0	-1.60171E-16	1
mmu-miR-741-3p	0	0	-1.60171E-16	1
mmu-miR-741-5p	0	0	-1.60171E-16	1
mmu-miR-742-3p	0	0	-1.60171E-16	1
mmu-miR-742-5p	0	0	-1.60171E-16	1
mmu-miR-743a-3p	0	0	-1.60171E-16	1
mmu-miR-743a-5p	0	0	-1.60171E-16	1
mmu-miR-743b-3p	0	0	-1.60171E-16	1
mmu-miR-743b-5p	0	0	-1.60171E-16	1
mmu-miR-7578	0	0	-1.60171E-16	1
mmu-miR-758-5p	0	0	-1.60171E-16	1
mmu-miR-759	0	0	-1.60171E-16	1
mmu-miR-760-3p	0	0	-1.60171E-16	1
mmu-miR-760-5p	0	0	-1.60171E-16	1
mmu-miR-761	0	0	-1.60171E-16	1
mmu-miR-762	0	0	-1.60171E-16	1
mmu-miR-763	0	0	-1.60171E-16	1
mmu-miR-764-3p	0	0	-1.60171E-16	1
mmu-miR-764-5p	0	0	-1.60171E-16	1
mmu-miR-7647-3p	0	0	-1.60171E-16	1
mmu-miR-7647-5p	0	0	-1.60171E-16	1
mmu-miR-7649-5p	0	0	-1.60171E-16	1
mmu-miR-7650-3p	0	0	-1.60171E-16	1
mmu-miR-7651-3p	0	0	-1.60171E-16	1
mmu-miR-7652-5p	0	0	-1.60171E-16	1
mmu-miR-7656-3p	0	0	-1.60171E-16	1
mmu-miR-7657-3p	0	0	-1.60171E-16	1
mmu-miR-7657-5p	0	0	-1.60171E-16	1
mmu-miR-7659-3p	0	0	-1.60171E-16	1
mmu-miR-7660-3p	0	0	-1.60171E-16	1

mmu-miR-7660-5p	0	0	-1.60171E-16	1
mmu-miR-7661-5p	0	0	-1.60171E-16	1
mmu-miR-7662-3p	0	0	-1.60171E-16	1
mmu-miR-7662-5p	0	0	-1.60171E-16	1
mmu-miR-7663-5p	0	0	-1.60171E-16	1
mmu-miR-7664-5p	0	0	-1.60171E-16	1
mmu-miR-7665-3p	0	0	-1.60171E-16	1
mmu-miR-7665-5p	0	0	-1.60171E-16	1
mmu-miR-7666-3p	0	0	-1.60171E-16	1
mmu-miR-7666-5p	0	0	-1.60171E-16	1
mmu-miR-7668-3p	0	0	-1.60171E-16	1
mmu-miR-767	0	0	-1.60171E-16	1
mmu-miR-7673-3p	0	0	-1.60171E-16	1
mmu-miR-7673-5p	0	0	-1.60171E-16	1
mmu-miR-7674-3p	0	0	-1.60171E-16	1
mmu-miR-7676-5p	0	0	-1.60171E-16	1
mmu-miR-7677-5p	0	0	-1.60171E-16	1
mmu-miR-7678-3p	0	0	-1.60171E-16	1
mmu-miR-7678-5p	0	0	-1.60171E-16	1
mmu-miR-7680-3p	0	0	-1.60171E-16	1
mmu-miR-7680-5p	0	0	-1.60171E-16	1
mmu-miR-7681-3p	0	0	-1.60171E-16	1
mmu-miR-7681-5p	0	0	-1.60171E-16	1
mmu-miR-7682-3p	0	0	-1.60171E-16	1
mmu-miR-7682-5p	0	0	-1.60171E-16	1
mmu-miR-7683-3p	0	0	-1.60171E-16	1
mmu-miR-7684-3p	0	0	-1.60171E-16	1
mmu-miR-7684-5p	0	0	-1.60171E-16	1
mmu-miR-7685-3p	0	0	-1.60171E-16	1
mmu-miR-7686-3p	0	0	-1.60171E-16	1
mmu-miR-7686-5p	0	0	-1.60171E-16	1
mmu-miR-7687-3p	0.361071183	0.196772266	-1.60171E-16	1
mmu-miR-7688-3p	0	0	-1.60171E-16	1
mmu-miR-7689-3p	0	0	-1.60171E-16	1
mmu-miR-7689-5p	0	0	-1.60171E-16	1
mmu-miR-770-3p	0	0	-1.60171E-16	1
mmu-miR-770-5p	0	0	-1.60171E-16	1
mmu-miR-7a-2-3p	0	0	-1.60171E-16	1
mmu-miR-7b-3p	0	0	-1.60171E-16	1

mmu-miR-802-3p	0	0	-1.60171E-16	1
mmu-miR-802-5p	0	0	-1.60171E-16	1
mmu-miR-804	0	0	-1.60171E-16	1
mmu-miR-8090	0	0	-1.60171E-16	1
mmu-miR-8091	0	0	-1.60171E-16	1
mmu-miR-8095	0	0	-1.60171E-16	1
mmu-miR-8099	0	0	-1.60171E-16	1
mmu-miR-8100	0	0	-1.60171E-16	1
mmu-miR-8101	0	0	-1.60171E-16	1
mmu-miR-8102	0	0	-1.60171E-16	1
mmu-miR-8104	0	0	-1.60171E-16	1
mmu-miR-8106	0	0	-1.60171E-16	1
mmu-miR-8107	0	0	-1.60171E-16	1
mmu-miR-8108	0	0	-1.60171E-16	1
mmu-miR-8109	0	0	-1.60171E-16	1
mmu-miR-8110	0	0	-1.60171E-16	1
mmu-miR-8115	0	0	-1.60171E-16	1
mmu-miR-8116	0	0	-1.60171E-16	1
mmu-miR-8117	0	0	-1.60171E-16	1
mmu-miR-8119	0	0	-1.60171E-16	1
mmu-miR-871-5p	0	0	-1.60171E-16	1
mmu-miR-873a-3p	0	0	-1.60171E-16	1
mmu-miR-873a-5p	0	0	-1.60171E-16	1
mmu-miR-873b	0	0	-1.60171E-16	1
mmu-miR-875-3p	0	0	-1.60171E-16	1
mmu-miR-875-5p	0	0	-1.60171E-16	1
mmu-miR-876-3p	0	0	-1.60171E-16	1
mmu-miR-876-5p	0	0	-1.60171E-16	1
mmu-miR-878-3p	0	0	-1.60171E-16	1
mmu-miR-878-5p	0	0	-1.60171E-16	1
mmu-miR-880-3p	0	0	-1.60171E-16	1
mmu-miR-880-5p	0	0	-1.60171E-16	1
mmu-miR-881-3p	0	0	-1.60171E-16	1
mmu-miR-881-5p	0	0	-1.60171E-16	1
mmu-miR-882	0	0	-1.60171E-16	1
mmu-miR-883a-3p	0	0	-1.60171E-16	1
mmu-miR-883a-5p	0	0	-1.60171E-16	1
mmu-miR-883b-3p	0	0	-1.60171E-16	1
mmu-miR-883b-5p	0	0	-1.60171E-16	1

mmu-miR-92b-5p	0	0	-1.60171E-16	1
mmu-miR-935	0	0	-1.60171E-16	1
mmu-miR-96-3p	0.722142365	0.393544532	-1.11022E-16	1
mmu-miR-9768-3p	0	0	-1.60171E-16	1
mmu-miR-9768-5p	0	0	-1.60171E-16	1
mmu-miR-9769-3p	3.610711827	1.967722658	0	1
mmu-miR-9769-5p	0	0	-1.60171E-16	1
mmu-miR-99a-3p	0	0	-1.60171E-16	1
