# Supplementary material for: Deletion of miR-150 Prevents Spontaneous T Cell Proliferation and the Development of Colitis
Source: Gastro Hep Adv. 2023 Feb 4;2(4):487–96. doi: 10.1016/j.gastha.2023.01.021 (PMC11308117; doi:10.1016/j.gastha.2023.01.021)
Supplement: Figure A1 [file mmc2.pdf]

Supplementary Figure 1

A

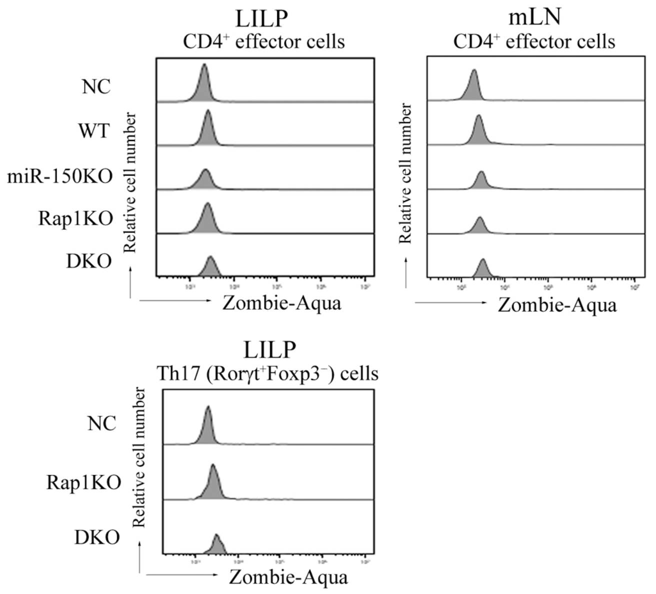

B

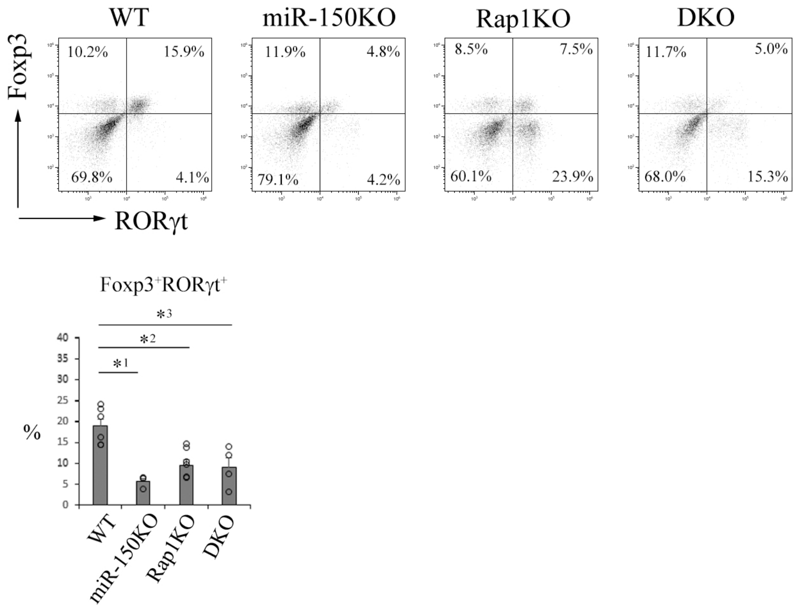

Figure A1 Apoptosis in effector CD4$^+$ cells and the ratio and number of RORγt$^+$ Treg cells were not increased in the LILP of DKO mice

(A) Representative flow cytometry profiles of zombie dye. (Upper) Apoptosis of effector CD4$^+$ cells derived from mLNs of WT, miR-150KO, Rap1KO and DKO mice was measured by the incorporation of zombie dye. (Lower) Apoptosis of Rorγt$^+$Foxp3$^-$ (Th17) cells derived from Rap1KO and DKO mice was measured by the incorporation of zombie dye.

(B)(Upper) Representative RORγt and Foxp3 profiles of effector CD4$^+$ cells from the LILP of WT, miR-150KO, Rap1KO, and DKO mice at 8–12 weeks of age. (Lower) The percentages of RORγt$^+$Foxp3$^+$ cells from the LILP of WT, miR-150 KO, Rap1KO, and DKO mice at 8–12 weeks of age (n = 4-7). Data represent the mean ± S.E.M. $*^1P < 0.001$, $*^2P < 0.003$, and $*^3P < 0.006$ compared with WT mice.
