# Supplementary material for: Deletion of miR-150 Prevents Spontaneous T Cell Proliferation and the Development of Colitis
Source: Gastro Hep Adv. 2023 Feb 4;2(4):487–96. doi: 10.1016/j.gastha.2023.01.021 (PMC11308117; doi:10.1016/j.gastha.2023.01.021)
Supplement: Figure A2 [file mmc3.pdf]

# Supplementary Figure 2

A

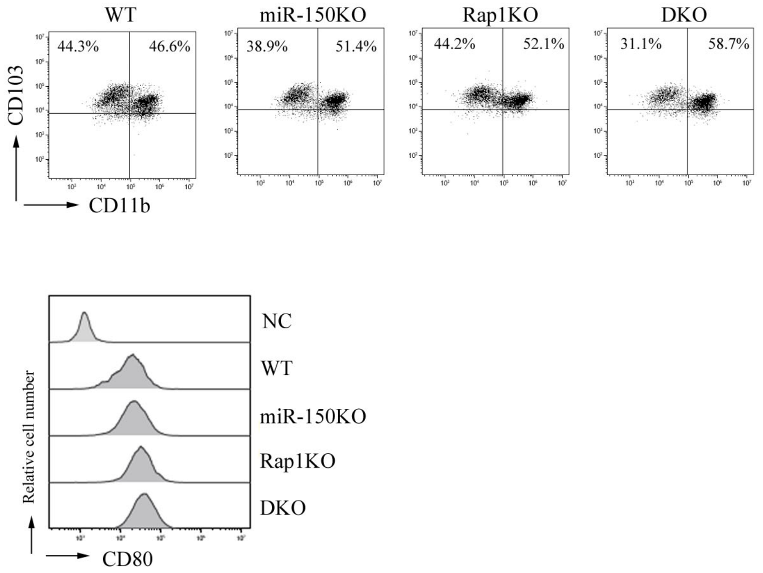


B

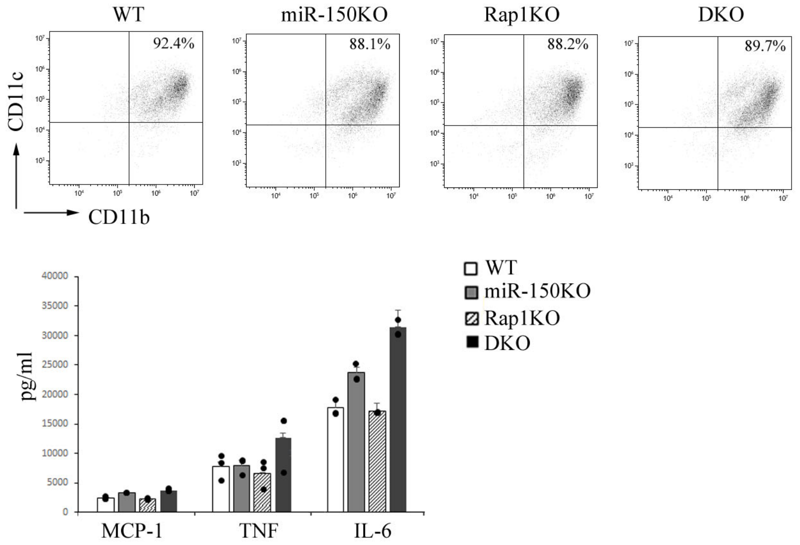


Figure A2 Effects of miR-150 on the subsets and cytokine production of dendritic cells.

(A)(Upper) Representative flow cytometry profiles of $CD103^{+}$ and $CD11b^{+}$ profiles of $CD11c^{+}$ $MHCII^{high}$ cells from the mLN of WT, miR-150KO, Rap1KO and DKO mice at 8-12 weeks of age. (Lower) Representative flow cytometry profiles of CD80 expressed on $CD11c^{+}MHC\ II^{high}CD103^{+}CD11b^{+}$ DCs in the mLNs of WT, Rap1KO, miR-150KO and DKO mice.

(B) (Upper) Representative flow cytometry profiles of $CD11c^{+}$ and $CD11b^{+}$ profiles of bone marrow-derived dendritic cells (BMDCs) derived from WT, miR-150KO, Rap1KO and DKO, as described in the Methods. (Lower) The amounts of Monocyte chemoattractant protein-1 (MCP-1), Tumor necrosis factor-α (TNF) and IL-6 in the supernatants of BMDCs from WT, miR-150KO, Rap1KO and DKO were measured in triplicate using Cytometric Bead Array (CBA) kit. Graphs represent the mean ± S.E.M.
