# Supplementary material for: Deletion of miR-150 Prevents Spontaneous T Cell Proliferation and the Development of Colitis
Source: Gastro Hep Adv. 2023 Feb 4;2(4):487–96. doi: 10.1016/j.gastha.2023.01.021 (PMC11308117; doi:10.1016/j.gastha.2023.01.021)
Supplement: Figure A3 [file mmc4.pdf]

Supplementary Figure 3

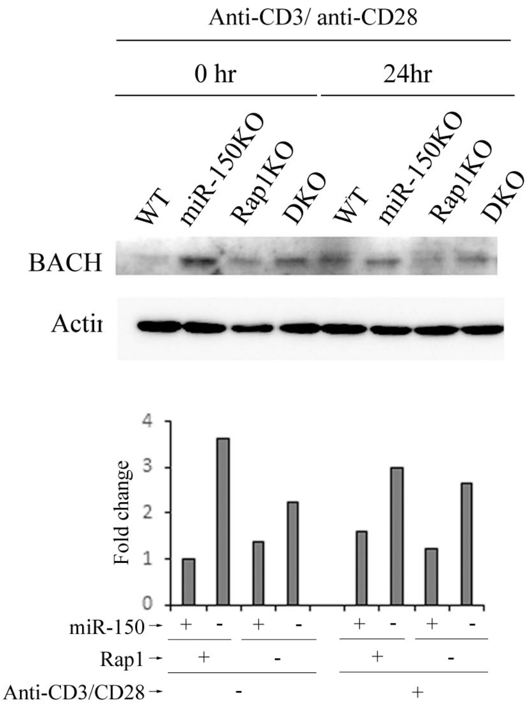

Figure A3 Effects of miR-150 deficiency on BACH2 expression.

(Upper) Naïve CD4$^+$ cells from WT, miR-150KO, Rap1KO and DKO mice were stimulated with 0.5 μg/ml of anti-CD3 in the presence of anti-CD28 for 24 hours. Total lysates from the stimulated cells were immunoblotted for anti-BACH2 and actin. (Lower) Quantification of BACH2 in the experiments above, which is presented as fold change of normalized abundance of BACH2 by the total amount of actin in CD4$^+$ cells before or after anti-CD3/CD28 stimulation relative to unstimulated WT CD4$^+$ cells (adjusted to 1).
